# The influence of thermal and hypoxia induced habitat compression on walleye (*Sander vitreus*) movements in a temperate lake

**DOI:** 10.1186/s40462-024-00505-6

**Published:** 2025-01-07

**Authors:** J. L. Brooks, E. J. I. Lédée, S. M. Larocque, S. J. Cooke, E. Brown, J. D. Midwood

**Affiliations:** 1 Department of Biology, Carleton University, 1125 Colonel By Drive, Ottawa, ON Canada; 2 Great Lakes Laboratory for Fisheries and Aquatic Science, Fisheries and Oceans Canada, 867 Lakeshore Road, Burlington, ON Canada; 3 Department of Biology, Institute of Environmental and Interdisciplinary Science, Carleton University, 1125 Colonel By Drive, Ottawa, ON Canada; 4 Lake Ontario Management Unit, Glenora Fisheries Station, Ontario Ministry of Natural Resources and Forestry, Picton, ON Canada

**Keywords:** Habitat compression, Hypoxia, Movement, Abiotic habitat

## Abstract

**Background:**

Globally, temperate lakes are experiencing increases in surface water temperatures, extended periods of summer stratification, and decreases of both surface and deep water dissolved oxygen (DO). The distribution of fish is influenced by a variety of factors, but water temperature and dissolved oxygen are known to be particularly constraining such that with climate change, fish will likely feel the “squeeze” from above and below.

**Methods:**

This study used acoustic telemetry to explore the effects of both thermal stratification and the deoxygenation of the hypolimnion on walleye (*Sander vitreus*) movements in a coastal embayment in Lake Ontario. Using historical water quality monitoring data, we documented seasonal and annual fluctuations in availability of both ‘suitable’ (all temperatures, DO > 3 mg/L) and ‘optimum’ (temperatures 18–23 °C, DO > 5mg/L) abiotic habitat for walleye and determined how these changes influenced walleye movements over a three-year period.

**Results:**

Hypoxia (< 3 mg/L DO) was present in Hamilton Harbour every summer that data were available (32 of the 42 years between 1976 and 2018), with a maximum of 68.4% of the harbour volume in 1990. We found that thermal stratification and a hypoxic hypolimnion greatly reduced the volume of suitable habitat during our telemetry study. The reduction of suitable habitat significantly reduced walleye movement distances, however as the summer progressed, this remaining suitable habitat warmed into their thermal optimum range which was found to increase walleye movement distances. Despite the seemingly poor conditions, tagged walleye remained in the harbour for most of the year, and were the fastest growing individuals compared to other sampled coastal subpopulations in Lake Ontario.

**Conclusions:**

Although we documented a reduction in the quantity of non-hypoxic habitat available to walleye, the water temperature of the remaining habitat increased throughout the summer into the physiologically optimum range for walleye and increased in metabolic quality. Many abiotic factors influence how, where, and what habitat fish choose to use, and this study reveals the importance of considering both habitat quality (temperature and dissolved oxygen) and quantity when evaluating fish habitat use and behaviour.

**Supplementary Information:**

The online version contains supplementary material available at 10.1186/s40462-024-00505-6.

## Background

Movement allows animals to select their surrounding environment and theoretically, the ultimate function of all types of movement is to increase an individual’s fitness [1]. Optimal movement strategies of an animal can vary in response to environmental (external) and physiological (internal) conditions [2, 3]. Ecosystems experience variability in resources (abiotic and biotic), predation risk, and intraspecific relationships [1]. Spatiotemporal distribution of resources dominates many ecological processes and the structure of resources can underpin movement strategies among a diverse range of taxa [1, 4–7].

Dissolved oxygen (DO) is an essential resource for all aquatic organisms that rely on water for respiration; however, its availability can fluctuate on a daily, seasonal, or annual basis. Levels of DO can be reduced because of natural and anthropogenic processes. For example, on a fine spatiotemporal scale, submerged aquatic vegetation can produce DO during the day via photosynthesis, and then consume DO during the night during respiration [8]. On a larger scale, many aquatic ecosystems will stratify thermally on a seasonal basis. During summer, any DO beneath the thermocline can be consumed by microbial activity and cannot be replenished until fall when the full water column reaches a similar temperature and mixing occurs [9]. Anthropogenic activities exacerbate the introduction of excess nutrients via agricultural practices [10] and urban development (e.g., sewage treatment infrastructure), which collectively can lead to eutrophication and low levels of DO, often called hypoxia (< 2 mg/L; threshold for various species) or anoxia (0 mg/L; [11]).

Organisms respond to changing DO and water temperature in a variety of ways, from physiological adaptations to behavioural avoidance and the thresholds at which they start to experience detrimental effects can vary (reviewed in [12]). These fluctuating levels of DO and water temperature, and the species-specific tolerances and responses play an important role in structuring aquatic ecosystems [13–15]. Fish are mobile and will often move away from sub-optimal conditions before undergoing physiological stress [12, 16, 17], however this avoidance behaviour can have implications far beyond the individual. Previous research has shown hypolimnetic hypoxia does indeed alter the availability of suitable habitat to a variety of marine and freshwater fish species, with the magnitude of their response dependent on their metabolic requirements and tolerance of low DO [18–21]. The reduction of habitat can influence fish on an individual level by forcing fish into habitat of suboptimal quality, which can be metabolically costly if temperatures are not ideal [22].

Habitat compressions can occur as a result of the behavioural avoidance of suboptimal habitat conditions [23, 24] and can lead to crowding, starvation, overfishing [25]. Habitat compressions can increase the overlap between predators and their prey, which often favours the species that can tolerate the lowest levels of oxygen [16]. Changes in distributions, abundance, and predator–prey dynamics influence other components of the food web including the fish community, and ultimately the whole aquatic ecosystem; therefore, understanding species responses to changes or reductions in abiotic habitat is critical.

Walleye (*Sander vitreus*) are a cool-water apex piscivore of economic and ecological importance in North America [26]. Understanding their habitat use, and how anthropogenic stressors such as hypoxia influence their habitat use is important for effective fisheries management. Walleye are one of the most tracked species in the Laurentian Great Lakes (Great Lakes Acoustic Telemetry Observation System, GLATOS https://glatos.glos.us/) and their habitat use has been shown to vary on a seasonal basis [27–31]. Temperature and light levels are thought to be the biggest contributor to this seasonal variation [31, 32], although many other abiotic and biotic factors change on a seasonal basis, including DO levels. The species-specific consequences of hypoxia in an aquatic ecosystem are not always negative [32] and, for walleye, spatial bioenergetic models of Growth Rate Potential (‘the expected growth rate for a fish of a particular age in a volume of water with known habitat conditions’; [32]) have shown that summer hypoxia may increase the *quality* of their habitat due to the compression of their prey into normoxic refugia [32]. Resources are not limitless, however, and inter- and intra-specific competition for these resources can lead to reduced fish growth [33]. The compression of prey may increase the foraging efficiency of walleye and reduce their search and foraging distances, however there is little understanding of how habitat compression may influence walleye movements in the wild.

To further explore the impact of oxythermal habitat compression on walleye, we tracked wild walleye movements for three years in a system that undergoes thermal stratification and seasonal hypoxia. More specifically, we quantify how the oxythermal habitat changes throughout the summer stratification period across five decades and explore how these changes influence walleye displacement distances over three years. We hypothesized that a hypoxic hypolimnion would reduce the amount of habitat available to walleye and reduce the distances walleye would move.

## Methods

### Study system

**Fig. 1:**
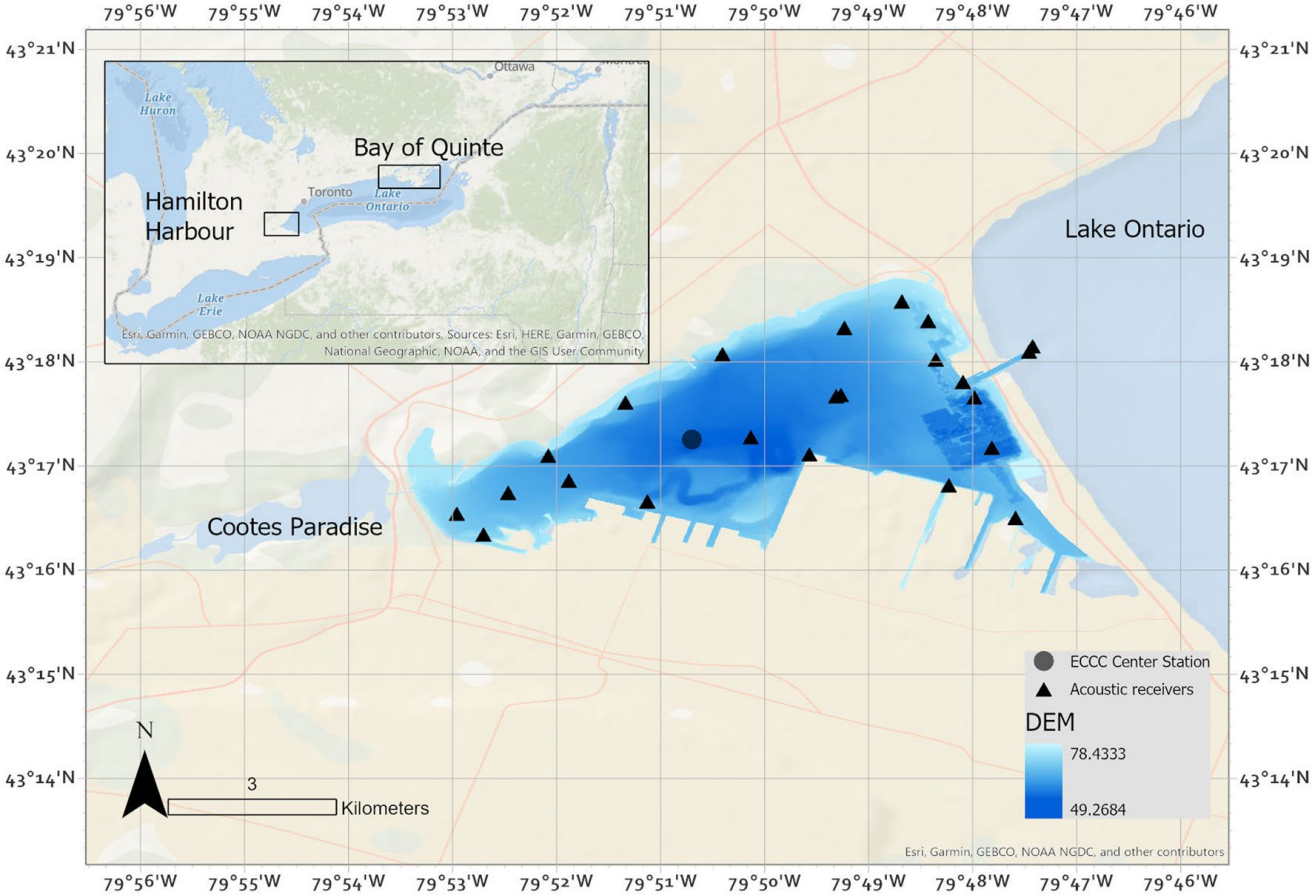
Hamilton Harbour at the west end of Lake Ontario. Acoustic receivers used in analyses are indicated with triangles, Environment and Climate Change Canada’s Center Station is indicated with a dot. Bathymetry is shown as a blue layer (a representation of the elevation of the lakebed above mean sea level, in meters), with Center Station located at the deepest part

This study was completed in Hamilton Harbour, a coastal embayment at the western end of Lake Ontario (43.30048 N, − 79.80591 W; Fig. 1) that has long been known to have issues with seasonal hypoxia [34, 35]. The western, northern, and north-eastern shoreline are characterized by rocky shorelines, shallow vegetated areas, and man-made rocky islands and shoals. The southern shoreline is characterized by harbour walls, two steel plants, and several marinas. The maximum depth in the Harbour is 25 m in the center, the mean depth is 13 m, and the surface area is 21 km^2^.

### Study species

Walleye are a cool-water piscivorous species with optimal temperatures ranging between 20 and 23 °C [36, 37], although free swimming walleye have been shown to use lower temperatures in the wild. Raby et al. [31] used temperature logging tags in walleye in Lake Erie and found they used water temperature of 18–23 °C in the summer. Minimum DO thresholds are typically determined as the value of DO when fish lose equilibrium in captive conditions and juvenile and adult walleye lose equilibrium at < 1.5 mg/L [38]. Previous field observations of free-swimming walleye have shown avoidance of DO levels of 3, 4, and 5 mg/L [39]. Walleye can see well in low light, and are known to be crepuscular in oligotrophic systems, and likely diurnal feeders during the day in more turbid systems [40–42]. Walleye were considered extirpated in Hamilton Harbour by the mid-twentieth century [43]. The walleye used in this study were likely stocked as fingerlings into Hamilton Harbour in 2012 by the Ontario Ministry of Natural Resources and Forestry (OMNRF) from a parent stock originating in the Bay of Quinte, 230 km east in Lake Ontario ([43], Fig. 1).

Fish size has been documented to influence migration distances [44], therefore the lengths of individual walleye were included in the analysis. Walleye have been routinely sampled with trapnets and their ages assessed using otoliths by OMNRF in Lake Ontario over the last two decades via their Near Shore Community Index Netting (NSCIN) program [43, 45]. In the present study, walleye were tagged throughout a 4-year period (2015–2018) and to account for their increasing size over the study period (and perhaps increasing displacement distances), age and fork length data for walleye (N = 1922) caught in Lake Ontario (as part of the NSCIN program) were used to develop a regionally specific von Bertalanffy Growth Function (VBGF) growth curve using r package ‘FSA’ ([46]; data obtained from OMNRF). The age of each walleye during their initial capture was estimated using the growth curve (in years), which were used to calculate their projected fork lengths (mm) for each of the subsequent years they were in the study (Supplementary Information Table 1). A Hamilton specific VBGF growth curve (Eq. 1) was calculated and plotted alongside all Lake Ontario samples to compare their growth curves to other sampling locations, however due to limited sample size, tagged walleye were assigned ages using the full Lake Ontario growth rate (Eq. 2).

**Table 1:** The first stage of model selection included the full data set from April to November, the second stage included only months when hypoxia was present in the Harbour and habitat compression occurred (stages compared separately with AIC)

Stage	Model #	Model equation	Deviance (%)	AIC
Full data (n = 140)	1	DD ~ s(Suitable) + s(FL) + s(Walleye ID, ‘re’) + s(Year, ‘re)	48.6	2505.9
	2	DD ~ s(Optimum) + s(Walleye ID, ‘re’) + s(Year, ‘re)	52.6	2477.5
Hypoxia present (n = 72)	3	DD ~ s(Suitable) + s(Walleye ID, ‘re’) + s(Year, ‘re)	87.4	1204.8
	4	DD ~ s(Optimum) + s(Walleye ID, ‘re’) + s(Year, ‘re)	79.9	1227.1
	5	DD ~ s(Optimum) + s(Suitable) + s(Walleye ID, ‘re’) + s(Year, ‘re)	92.0	1182.8

Model terms include walleye monthly displacement distance (DD), the amount of ‘suitable’ habitat (all temperatures, DO > 3 mg/L), the amount of ‘optimum’ habitat (temperature between 18 and 23 °C and DO > 5 mg/L), the fork length of the walleye (FL in mm, projected per year based on size at capture and an estimated age). Walleye ID and year of study were included as random effects (‘re’). The table also includes the deviance explained, and AIC score from the two stages of comparison

Equation 1: Hamilton Harbour walleye growth rate:

$${\text{FL}} = 642.5\left( {1 - {\text{e}}^{{ - 0.375({\text{age}} + 0.5)}} } \right)$$

Equation 2: Lake Ontario walleye growth rate:

$${\text{FL}} = 601.4\left( {1 - {\text{e}}^{{ - 0.31({\text{age}} + 1.006)}} } \right)$$

### Environmental variables

A digital elevation model was obtained from Fisheries and Oceans Canada ([47]; Supplementary Information Fig. 1) and used to calculate a hypsographic curve to determine the area (and subsequently the volume) for each 1 m depth of the Harbour, based on an average surface value above sea level of 75 m (Supplementary Information Table 2). Cootes Paradise marsh area was excluded as walleye have not been detected on any acoustic receivers inside the marsh, nor detected attempting to enter through a manned fishway (Fig. 1). All receivers used in the Network Analysis have been documented as used by walleye [27] and positioned within the depth ranges used by Hamilton Harbour walleye (Larocque et al. in review). All temperature and DO measurements were obtained from the Environment and Climate Change Canada’s Center Station, a dataset starting in 1976, containing daily measurements for spring-fall of 32 of the last 46 years (43.28755N, − 79.845W; Fig. 1; data provided by David Depew). We have previously documented intense variability in the temperature and DO over short time scales (hours and days) in the littoral areas of the Harbour because of the wind-driven oscillations of the thermocline [34, 39]. The thermocline at the center of the system near Center Station is the most stable (refer to Fig. 4 of [34]) and represents, on average, the depth of the thermocline throughout the Harbour. As the deployment and retrieval of equipment varied yearly, annual data were subset to only include data collected between 27 May and 2 October to ensure data throughout each summer were comparable. Measurements at Center Station were made at approximately every 1-m from the surface to a depth of 24 m using a YSI profiling buoy. The profiler recorded DO, temperature, specific conductance, pH, and depth every 120 min [48]. Each 1 m recording was given a habitat category based on the temperature and DO value. If DO was above 3 mg/L, it was categorized as ‘suitable’ habitat (after [37]). If DO was above 5 mg/L and the temperature was within the field-observed and physiologically optimum range of 18–23 °C, it was categorized as ‘optimum’ habitat. Each 1 m depth interval was assigned a volume from the hypsographic curve, and the total volume for each habitat category was calculated per day of year, and then averaged for each month of the study. Temporal trends in hypoxia and optimum habitat were explored by repeating this for all available historical data. The minimum, maximum, and mean proportions of each habitat category were calculated and plotted over time.

**Fig. 2:**
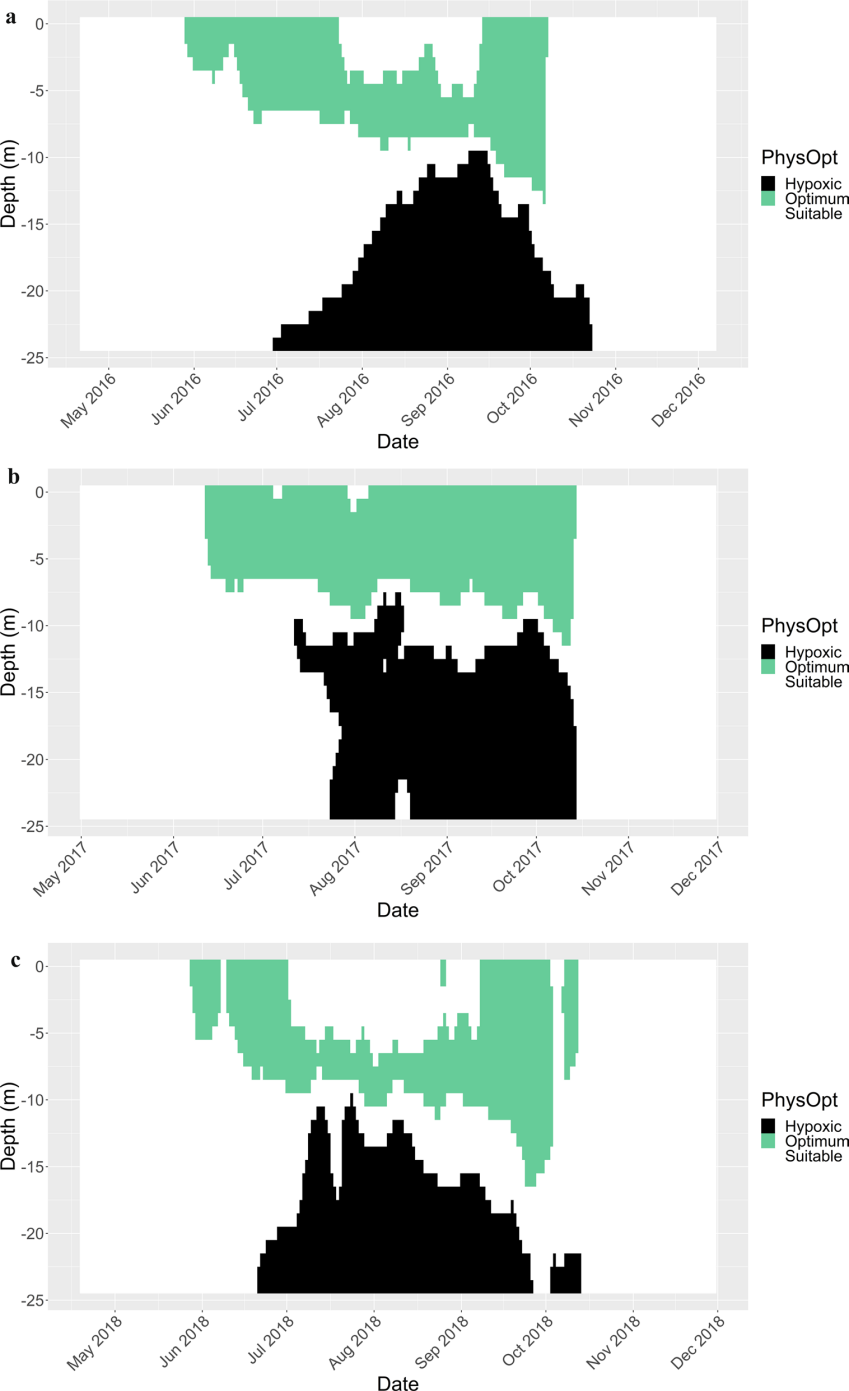
Categorized abiotic conditions per 1 m depth for each day of the year for **A** 2016, **B** 2017, and **C** 2018. Black shading is hypoxic (< 3 mg/L), green represents physiologically optimal for walleye (*Sander vitreus*; 18–23 °C, > 5 mg/L), and white is suitable (all temperatures, > 3 mg/L)

During the three-year telemetry study (2016–2018), temperatures ranged from 3.9 to 25.8 °C at the surface and DO ranged from 3.1 to 14.3 mg/L at the surface and 0.2–13.5 mg/L at the bottom (24 m below surface Fig. 2a–c). The water volume of the harbour during average water levels was 283 × 10^6^ m^3^. The volume of suitable habitat (all water temperatures, DO levels over 3 mg/L) ranged from the entire harbour during spring and fall to a minimum of 48 × 10^6^ m^3^ during August 2017 (a proportion of 17%; Fig. 2b).

### Fish telemetry

Walleye were captured during multiple sampling seasons between August 2015 and October 2018 (Supplementary Information Table 1), by either trap nets set as part of the OMNRF’s NSCIN surveys or an electrofishing boat (Smith-Root electrofishing boat model SR 18.EH; 250 V and 7 A). Tagging methods can be found in Supplementary Information.

Fish detection data for this study were obtained from 21 acoustic telemetry receivers (InnovaSea, VR2W 69 kHz, Bedford, Nova Scotia) deployed throughout the harbour between 2015 and 2021 (Fig. 1; Supplementary Information Table 3). Receivers were retrieved, serviced, and redeployed in the spring and fall of each year. Receiver data from 2016, 2017, and 2018 were used for this study. Residency was calculated simply by determining if each individual was detected within the harbour array or the Lake Ontario receivers per month and presented as a percentage. A total of 8.5 million walleye detections from 57 individuals were obtained and filtered down to 35 live walleye to be used in this study. Fish sizes at tagging ranged from 410 and 621 mm fork length, and projected fish sizes ranged between 450 and 621 mm fork length (Supplementary Information Table 1).

### Data processing

Detection data were sorted, split into individual transmitter ID files, and plotted on a ‘per fish’ basis to visually check for and remove dead fish, expelled transmitters, or erroneous transmitter IDs from the database. For each transmitter, detection data that met the criteria for false positive detections were removed from our analyses (by eliminating detections for which the minimum time between the last or next detection for the same transmitter on the same receiver was greater than 30 × the average tag delay of 120 s; [49]). Detections that occurred earlier than the minimum transmitter pinging interval across all receivers were also removed to reduce the risk of inflating the presence of fish in an area with a high degree of overlap in acoustic range (refer to Supplementary Information for more detailed methods).

The performance of acoustic telemetry can be affected by a variety of abiotic and biotic factors [50]. Unaccounted variation in detection efficiencies can interfere with conclusions drawn from telemetry data, in particular when determining seasonal changes in animal behaviour and the detections are affected by environmental conditions that change on a temporal basis [51]. Previous research in the harbour has determined that walleye space use is reduced during the thermal stratification period [27]. Subsequent research has also shown thermal stratification (and the varying densities of water) can influence the speed of sound, and therefore the ability of receivers to detect a transmission [52]. Wells et al. [52] modelled this relationship using a mathematical formula (Bellhop model) and determined the field observation data aligned with the model predicted distances. It is essential to account for this reduction in performance in analyses and interpretation. We therefore predicted a mean detection range (the distance at which 50% of the transmissions were successfully detected and recorded) for the 21 receivers during the stratified and isothermal time periods (Supplementary Information Fig. 2, Table 3). Predicted detection ranges fluctuated by a maximum decrease of 55.7% between isothermal and stratification across the entire study array of 21 receivers (total isothermal coverage = 9.36 km^2^, total stratified coverage = 5.21 km^2^; Supplementary Information Table 3).

The least-cost paths were calculated between each of the 21 receivers using the R package ‘rgdal’ [53]. Horizontal distances were calculated ‘as the walleye swims’ as opposed to ‘as the crow flies’ using coastal perimeters imported as shapefiles (Supplementary Information Fig. 3; shapefile obtained from Fisheries and Oceans Canada).

### Network analysis

A network analysis was conducted to determine movement within the harbour using R package ‘iGraph’ [54] for each individual walleye, per month (April to November), per year (2016, 2017, 2018) (methods followed [55–57], refer to Supplementary Information for detailed methods; Supplementary Information Fig. 4A and B; Tables 4–6).

The Network Analysis provided an edge list table containing the number of movements the walleye made between each node combination. To account for changes in detection ranges across stratified and isothermal conditions, we calculated an adjustment factor for every receiver pairing based on the mean detection ranges ([52]; Supplementary Information Table 3) using the steps presented in the Supplementary Information. We calculated seasonally weighted displacement distance per individual walleye between receiver A and B per month using this formula:

Total distance for receiver A and B = Number of movements between A and B * Stratified or isothermal adjustment factor between A and B * Least-cost distance between receiver A and B.

We totaled all displacement distances for each receiver combination to get a seasonally adjusted displacement distance value per walleye, per month, per year (Supplementary Information Table 7). These displacement distances are not intended to reflect definite swimming distances travelled by walleye, just the distances between the nodes and a means to compare *relative* movement in the harbour. The exact position of a transmitter cannot be determined accurately by a single receiver and the fish could be detected up to approximately 350 m either side of each receiver, therefore the actual distance travelled by a walleye from receiver to receiver is approximated.

Network Analysis was conducted on thirty-five fish resulting in 252 walleye networks during the time periods that had environmental data, and which made it through the 75% minimum monthly residency filter. Only 140 of those networks were deemed significantly different from the randomly generated networks and used in all further analyses, with a final fish count of thirty-two walleye (Supplementary Information Tables 4–6). The path weights for all 140 networks were compiled and overlayed onto a map of Hamilton Harbour to visualize where and how walleye movements change throughout the study period.

### Statistical analysis

To test the hypothesis that a reduction of suitable and optimum abiotic habitat would reduce walleye displacement distances, individual walleye monthly displacement distance was first plotted against the mean monthly volume of ‘suitable’ habitat (all temperatures, DO over 3 mg/L). Generalized Additive Mixed Models (GAMMs) were used to test the hypothesis due to non-linearity and the hierarchical nature of the dataset. Individual walleye monthly distances were modelled against the volume of suitable habitat and/or optimum habitat (18–23 °C, DO > 5 mg/L) that was available to them. The projected fork length of each fish was initially included as a potential predictor variable. Transmitter ID and year were assigned as random factors to account for the lack of independence with data points, individual variability in behaviour, and interannual variability in environmental conditions. As movement distances cannot be negative, a log link function was used to keep the intercept above zero.

Due to the number of potential combinations of explanatory variables (fork length, suitable habitat volume, optimum habitat volume—without interactions), models were built in steps. Stepwise backward selection was implemented [58, 59] and models were compared for their complexity and performance of explaining the variability in walleye displacement distances and the optimal sub-model was chosen based on the lowest AIC [60, 61]. The first set of models explored the movement data from the full study period and initially included fish fork length, the next set of models only explored data from months when hypoxia was present (compared with AIC separately from the first set of models). There was no relationship between fork length and walleye movements and fork length was removed from further models (Supplementary Information). All GAMMs were inspected using ‘gam.check’, autocorrelation was inspected using acf plots, concurvity (equivalent of collinearity in linear models) of the predictor variables was checked, and models were visually reviewed using residual plots and Q-Q plots (all exported into the Supplementary Information). The optimum and suitable habitat categories were calculated from the same body of water and concurvity plots showed a potential non-linear relationship (Supplementary Information). The model containing both suitable and optimum habitat categories as predictor variables of walleye movement was excluded from the final results and discussions, however the output is printed in the Supplementary Information.

## Results

### Environment

**Fig. 3:**
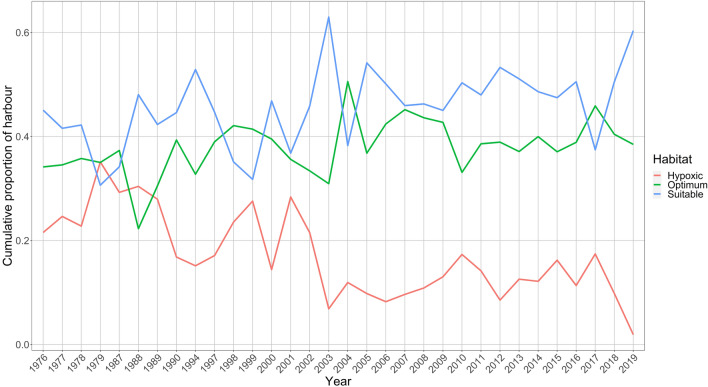
Proportion of the harbour per habitat category. Annual data were subset to the date range of 27 May to 02 October each year to account for varying deployment and retrieval dates each year. Daily volumes of each habitat category were summed, then divided by 129 days of available habitat to produce one cumulative proportion of each habitat category per year. Hypoxic habitat (red) is < 3 mg/L of DO and all water temperatures, suitable habitat (blue) is > 3 mg/L and all water temperatures, and physiologically optimum habitat (green) is > 5 mg/L and water temperatures between 18 and 23 °C

The volume of walleye optimum habitat (18–23 °C and > 5 mg/L DO) ranged between 0 and 194 × 10^6^ m^3^ (a proportion of 69%; September 2018, Fig. 2c) across the period 1976–2018. The categorization of each 1 m water depth strata per day between 27 May and 2 October was completed for all available data between 1976 and 2019 (Supplementary Information Table 8 and Fig. 5). Hypoxia was present in the Harbour every year that data were available, with a minimum daily proportion of the Harbour that was hypoxic of 0.08% (15 of the 32 years), and a maximum daily proportion of 65.9% (occurred in 1979 and 1988). The minimum daily proportion of the Harbour that was considered optimum for walleye was 5.9% (during 2001 and 2018) and the highest proportion was 91.3% (during 2018). The daily volumes of each habitat category were summed for each season and divided by the cumulative available volume for the season (129 days * 283 × 10^6^ m^3^) and showed a general decline of hypoxia over time and increasing trend in optimum and suitable habitat (Fig. 3).

### Walleye growth

**Fig. 4:**
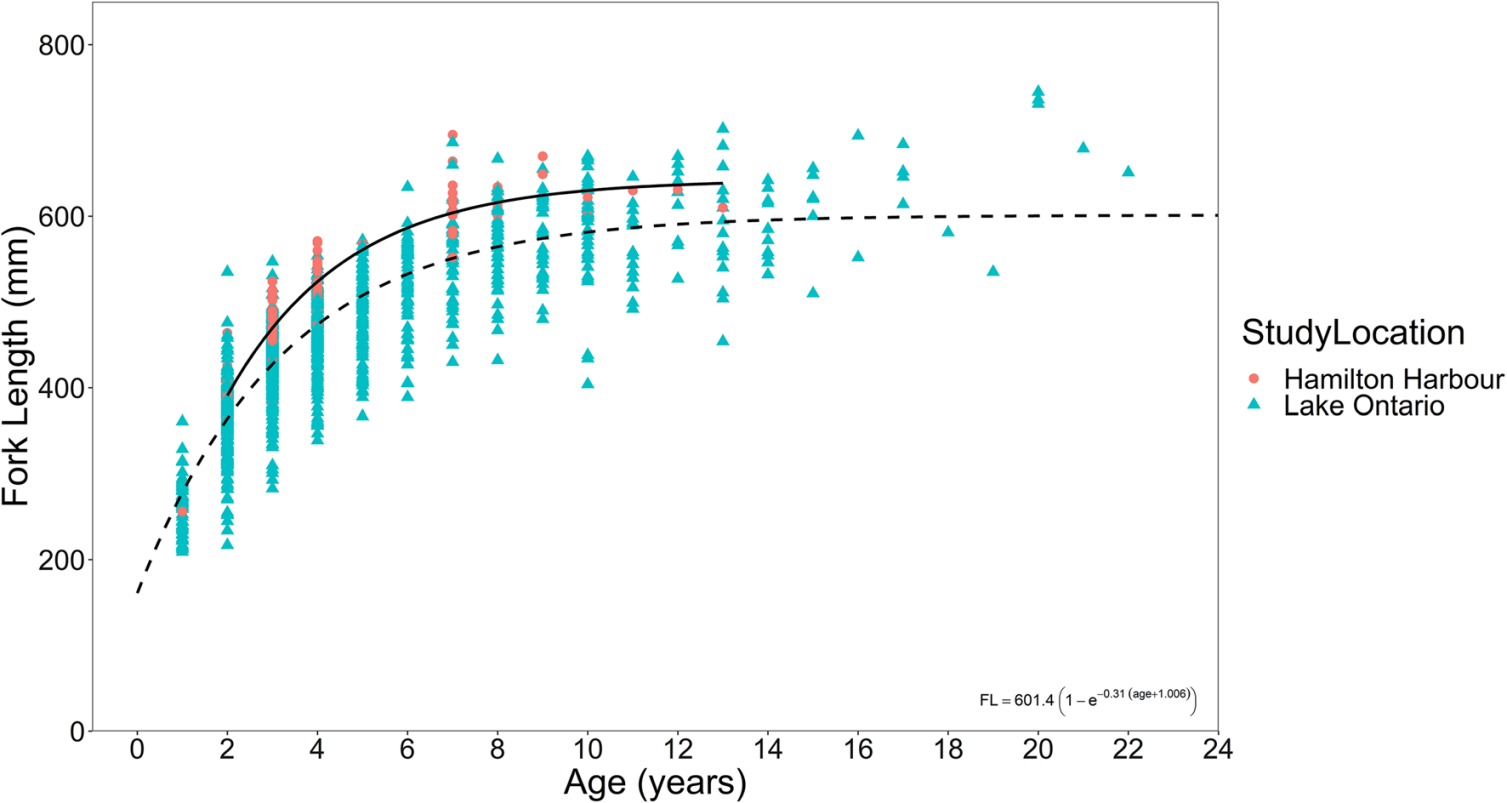
Age and fork length of walleye (*Sander vitreus*) sampled in western Lake Ontario by Ministry of Natural Resources and Forestry’s Nearshore Community Index Netting Survey (data provided by OMNRF). Pink circles and solid line indicate the lengths and growth rate for walleye sampled in Hamilton Harbour, respectively

Growth rate analysis from all sampled coastal Lake Ontario walleye subpopulations showed Hamilton Harbour walleye subpopulation as growing the fastest (*K* = 0.375 for Hamilton Harbour, 0.31 for Lake Ontario; Fig. 4). The caveat was that the NSCIN sampling efforts did does not include the larger walleye that typically migrate out of the Bay of Quinte (one of the monitored coastal subpopulations and the source walleye stock for Hamilton Harbour reintroduction efforts) during the time of sampling.

### Walleye movement

**Fig. 5:**
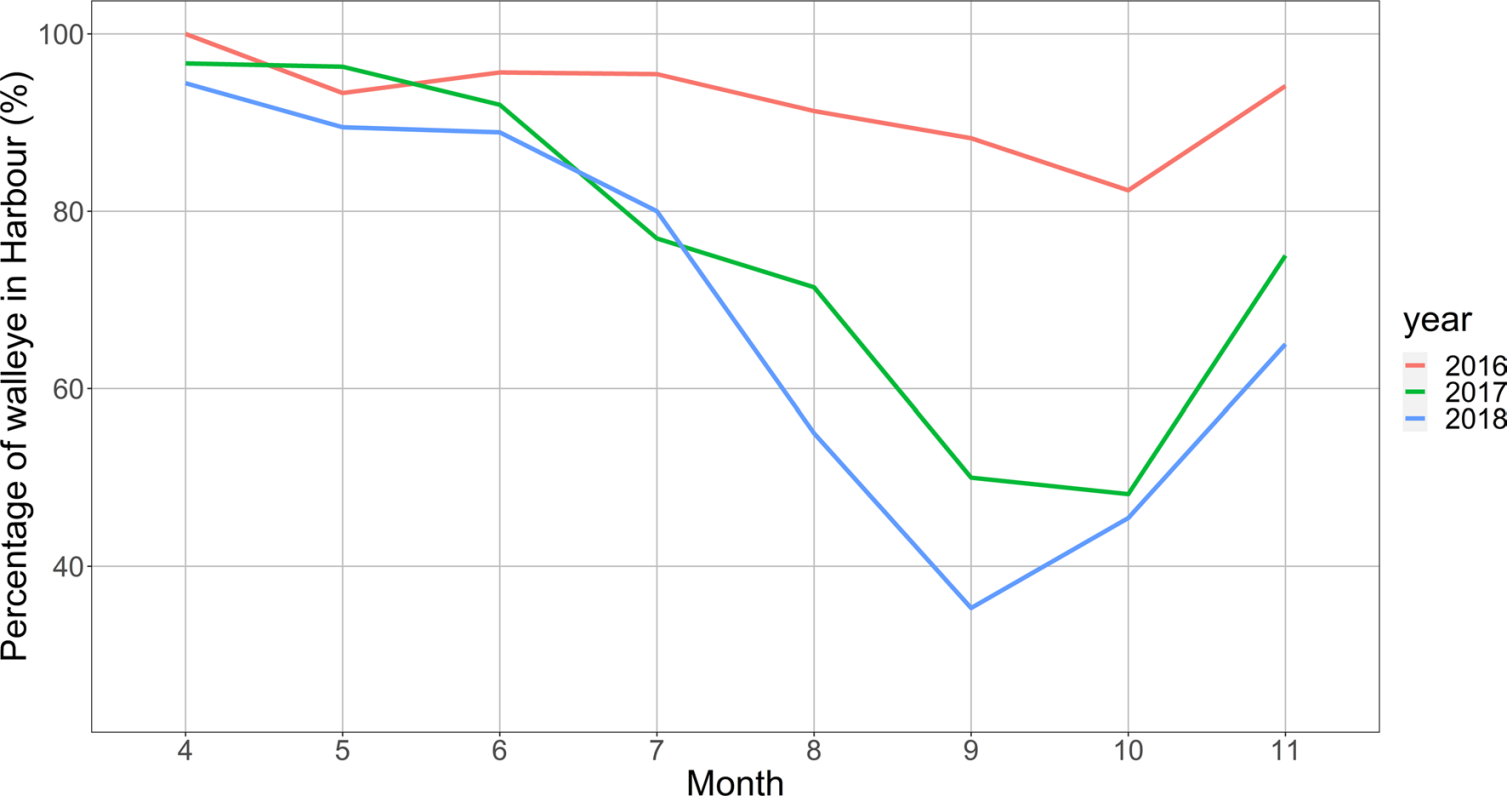
Percentage of tagged walleye (*Sander vitreus*) inside Hamilton Harbour per month for 2016 (red), 2017 (green), and 2018 (blue)

Residency within the Harbour varied greatly. The maximum proportion of tagged walleye that were detected within the Harbour was during April 2016 (100%), and a minimum proportion seen in September 2018 (35%, Fig. 5).

**Fig. 6:**
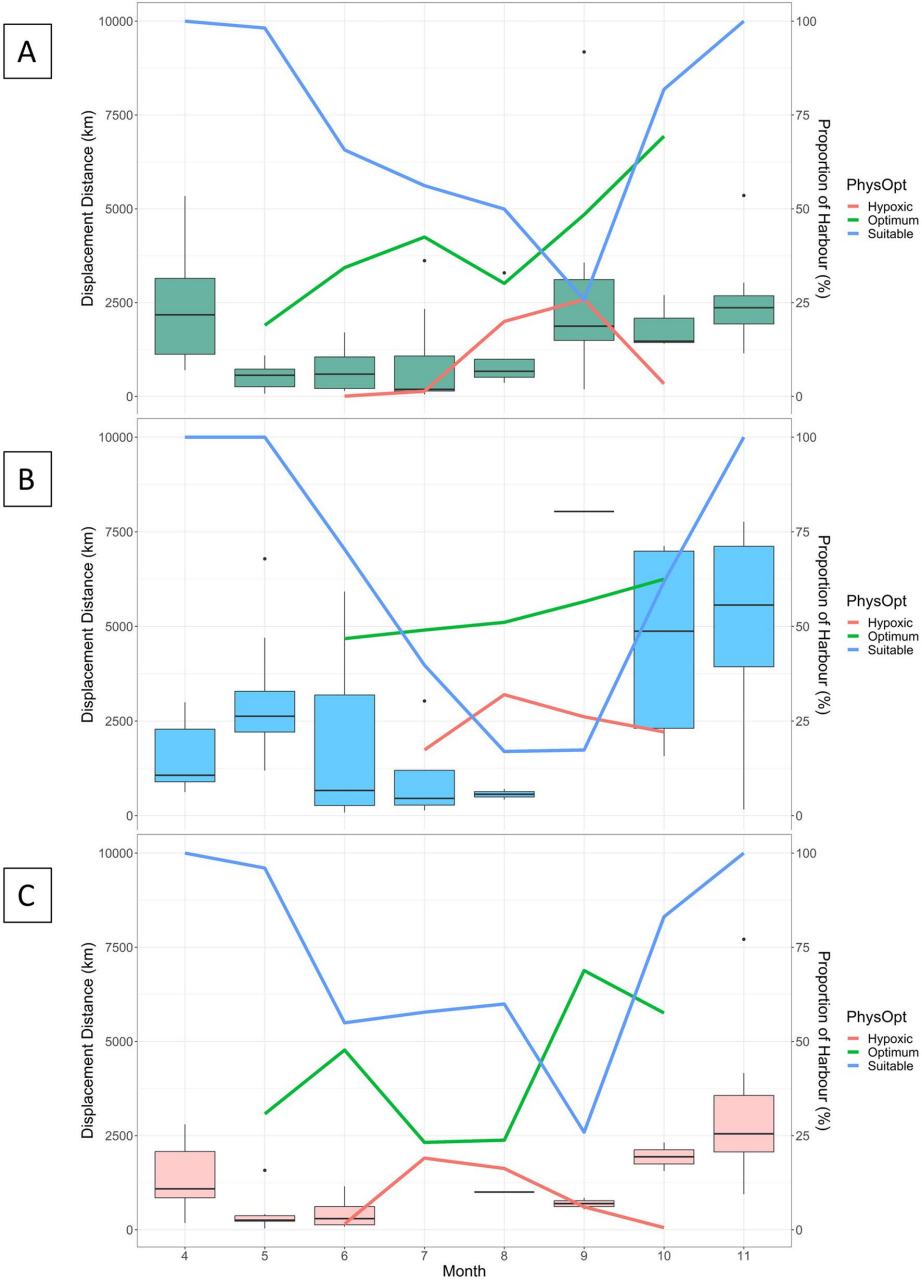
Monthly displacement distances (km) for all tagged walleye (*Sander vitreus*) and the proportion of the Harbour for each three habitat categories for **a** 2016, **b** 2017, and **c** 2018

**Fig. 7:**
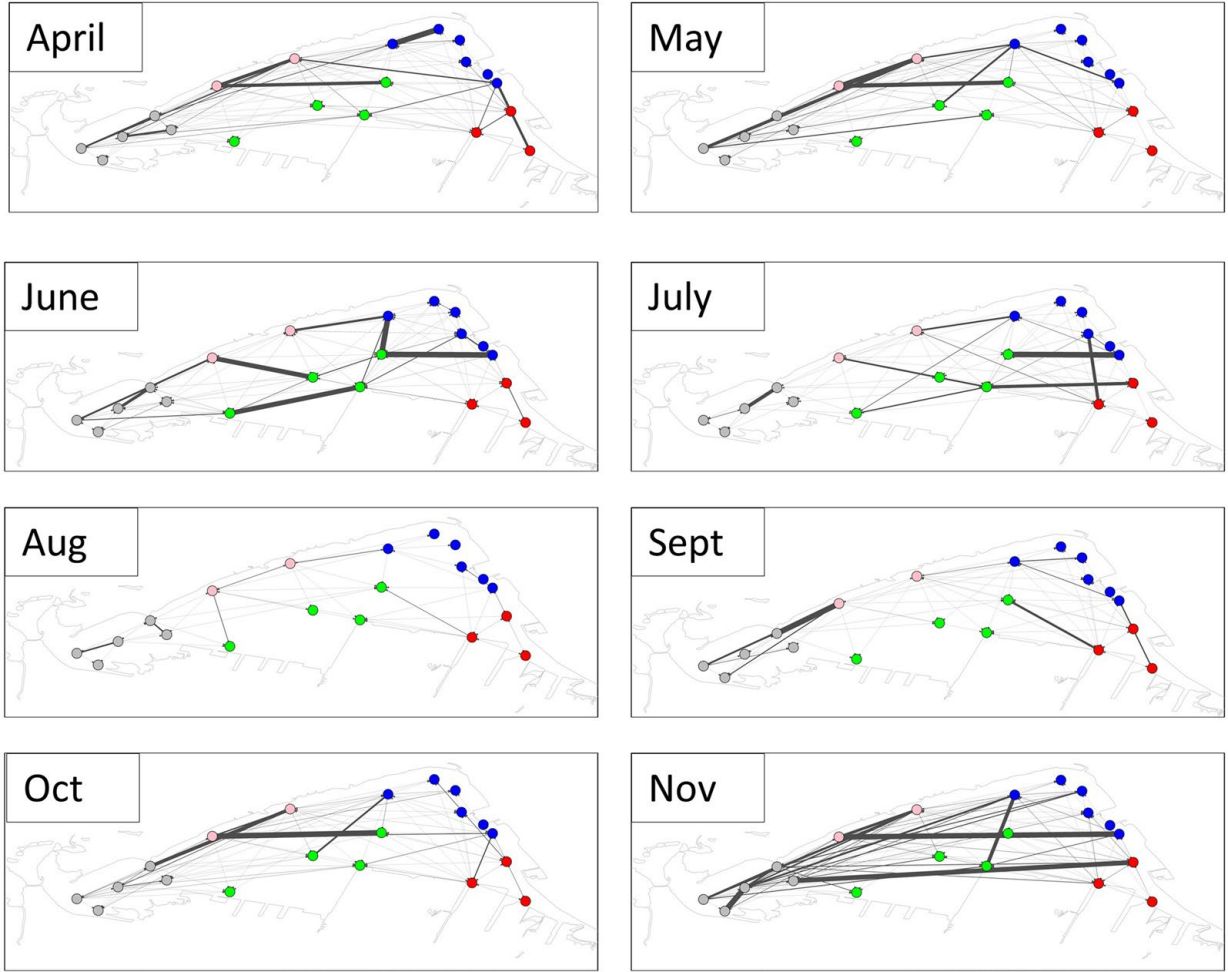
Monthly networks for all tagged walleye across all years. Thickness of connecting lines indicates the number of movements between those two nodes (receiver stations). Node colours indicate geographic region of the Harbour

Walleye monthly displacement distances ranged from 37.1 km to 9180.0 km, with the maximum observed during September 2016 (Fig. 6; Supplementary Information Table 7) and the minimum during May 2018. Monthly trends appeared similar across all the years, with a general decrease of movement from spring to the summer months, then an increase in the fall. Maps of the network path weights showed some variability month to month in how the walleye used the Harbour (Fig. 7), with the north shore appearing to be most popular in April and May and a noticeable reduction in movement in July and August. Walleye appeared to move extensively and used the whole Harbour in November (Fig. 7).

**Fig. 8:**
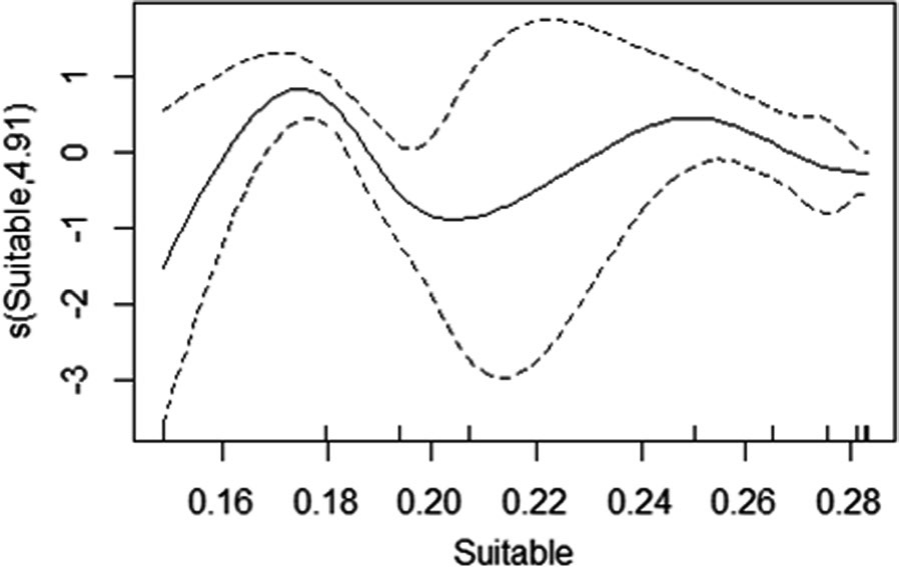
Model 3 output plots for walleye displacement distances vs proportion of the harbour volume that was Optimum or Suitable

Walleye movements throughout the full study period were influenced by the proportion of suitable and physiologically optimum habitat in Hamilton Harbour. For months when hypoxia was present in the system, the proportion of suitable habitat best described the displacement distances (88% deviance explained; Tables 1 and 2; Fig. 8; Supplementary Information). However, optimum habitat was also a significant predictor (*P* < 0.001, 80% deviance explained; Supplementary Information). Interestingly, displacement distance increased consistently with an increasing proportion of optimum habitat. The displacement distance also increased with an increasing proportion of suitable habitat but plateaued when suitable habitat proportion reached approximately two thirds of the harbour (Fig. 8). Individual ID and year were significant in all models indicating that there was inter-individual variability in movement behaviour beyond the length of the fish, and that there are likely unmeasured variables in the system that vary on an annual basis that also influence walleye movements. Walleye size was not a significant predictor of movement (Supplementary Material).

**Table 2:** Outputs for model 3 that was derived for just the months when hypoxia was present (i.e., stratified period) and included smoothing terms for Optimum and Suitable habitat volume as well as random effects for Walleye ID and Year

Model #	Parameter	Estimate	Std. error	*t* value	*P*
3	Intercept	6.9796	0.4449	15.69	< 0.0001
Smother terms					

Model #	Parameter	edf	Ref.df	*F*	*P*
3	s(Suitable)	4.912	5.294	5.944	< 0.001
	s(Year)	1.642	2	577.404	< 0.001
	s(Walleye ID)	19.106	29	20.794	< 0.0001
R-sq. (adj) =	0.803				
Deviance explained =	87.4%				
REML =	623.84	Scale est. =	7.7132e + 05	n =	72

## Discussion

The combination of environmental monitoring and telemetry provided insight into the complex relationship between hypoxia and habitat availability, and how this relationship can influence walleye residency and movement. Hamilton Harbour, like many temperate, eutrophic lakes, stratifies in the summer and the dissolved oxygen in the hypolimnion gradually decreases to below tolerable levels to most fish species. As the hypolimnion becomes more hypoxic, the volume of suitable habitat is compressed from below and we predicted a subsequent reduction in walleye movement (displacement distances). Our results supported this prediction; however, the relationship was non-linear and was complicated by water temperatures. As the volume of unsuitable habitat available for walleye increased, a proportion of the remaining suitable habitat increased in temperature into the physiologically optimum range for walleye (optimum habitat). When we explored the influence of the optimal habitat volume on walleye displacement distances, we found a significantly positive linear relationship. This indicated that although habitat quantity was decreasing throughout the summer months, habitat quality (metabolically) was gradually increasing and likely explained the complex non-linear relationship, i.e., as suitable habitat was decreasing in volume, movement decreased until the remaining habitat warmed into their thermal optimum range, when their movement subsequently increased.

Previous studies have explored seasonal trends in walleye residency and habitat use and determined the amount of space used by walleye in the summer months was the lowest of all seasons [27]. We predicted, therefore, that walleye would travel shorter distances in the summer months—potentially due to their own physiological tolerance restricting their movements, or the movements of their prey and therefore reducing the need for walleye to swim large distances to forage. Walleye displacement distances did vary throughout the three sampling seasons, and in support of previous findings, walleye moved the least during the summer months. This study explored walleye movements at a finer, monthly temporal scale than Brooks et al. [27], which revealed that summer is complicated with environmental and movement trends changing within the season. Our analysis revealed the contrasting relationships of an initial decrease in movement as hypoxia increases, to an increase in movement when the water temperatures reach physiological optimum. These within-season nuances were hidden in previous analyses, and we would therefore recommend other assessments of environmental influences on fish populations to consider a finer temporal scale than ‘season’.

Hypoxia is assumed to be detrimental to fish. Walleye had the ability to leave the system but largely remained resident to the Harbour during the hypoxic period, suggesting the hypoxia was not disadvantageous for them. The increased growth rates in the Harbour compared to other subpopulations also suggested walleye had sufficient prey and optimal temperatures for growth within the system. Our results are consistent with some aspects of the Growth Rate Potential model presented in Brandt et al. [32]. Their study concluded that although hypoxia reduced the volume of habitat available to walleye, their prey was concentrated into a narrower epilimnion, which increased foraging efficiency, and had optimal temperatures for walleye growth. Therefore, although there was hypoxia present, it was not detrimental to this species as far as growth rate potential. Their study differed from ours in that September was predicted to have the highest volume of high-quality habitat in terms of prey biomass and water temperatures while experiencing the lowest DO levels at the same time. The volume of high-quality habitat was predicted as almost twice as high during this time than either before or after the September hypoxia period [32]. Our study found that October and November had the greatest volume of optimum habitat for walleye.

Prey movements were not included in our study, however previous research into the fish community has shown seasonal variability in abundance and spatial distribution around the harbour. Previous hydroacoustic survey data in Hamilton Harbour in 2016 showed the seasonal distribution of prey fish density and biomass was greatest in the summer in the western portion of the Harbour [62]. This spatial concentration of forage fish in the west combined with walleye avoidance of hypoxic hypolimnetic waters could explain the reduced movements during the summer season. In the fall, however, prey fish density and biomass were comparable or higher across the whole system relative to the summer and were more evenly distributed spatially (particularly for comparable surveys completed in fall 2018; [63]). This evenly distributed prey biomass coincided with the deepening of the thermocline and thus reduced habitat compression for these fishes [62]. The coincident increase in walleye movements in fall would thus be consistent with a need for increased movement to forage effectively (as the prey is now less concentrated); however, we cannot discount the role of increased habitat availability in the observed increase in fall movement.

Hamilton Harbour walleye grew at a faster rate than all other subpopulations sampled in coastal systems of Lake Ontario by NSCIN [28]. Both Hamilton Harbour and the Bay of Quinte have been characterized as sheltered embayments [64]. Research into the phytoplankton communities and nutrient regimes (chlorophyll a and total phosphorus concentrations) have shown the two embayments to be comparable (Hamilton Harbour and Bay of Quinte) and higher than Toronto Harbour [65]. A potential explanation for the higher growth rates in Hamilton Harbour versus the rest of Lake Ontario subpopulations is the potential for an increased foraging efficiency as their prey may be compressed, and the availability of water temperature deemed optimum for growth. Larger walleye that spawn in Bay of Quinte during the spring often migrated out to the eastern basin/Lake Ontario proper and are therefore missed in the NSCIN efforts [28] and could have a different growth rate to the more resident, coastal individuals. As the walleye subpopulation have been reintroduced into the system after being absent for several decades (from Bay of Quinte stocks; [41]), the founder effect [66] may also explain these increased growth rates as the subpopulation may be founded from a few individuals that had naturally higher growth rates. Another potential explanation is the subpopulation is relatively young (2012 was considered the most successful year for stocking of fingerlings; [67]), and, as the Harbour generally had low piscivorous species abundance beforehand [68, 69] there may still be an imbalance between the predator and prey communities. Year was included as a random effect in each model and accounted for a significant amount of variation in all models, outside of temperature and DO, suggesting there are other drivers of walleye movement that vary on an annual basis. We would therefore recommend further exploration of the relationship between walleye habitat use and the spatiotemporal characteristics of the prey community.

Interestingly, another significant predictor of walleye displacement distance was the individual walleye but not as a result of their size. This could indicate there are some different migratory behavioural types that occur within the Hamilton walleye subpopulation. Partial migration (reviewed in [70]) has been explored in Lake Superior walleye [71]. Their study found migratory walleye were slightly larger than residents and predominantly female; however, size was not a significant predictor of variability in displacement distances in our study and individual sex was not available for our tagged fish. Other studies have shown size-based differences in habitat use in response to hypoxia and suggested larger fish secure the more oxygenated locations [72]. Further research into individual walleye movements and the extent of their migrations outside of the Hamilton array are recommended to explore the potential influence of size, individual personalities, and sex as drivers of migratory behaviour.

Understanding the drivers of movement of a commercially and recreationally fished species is important for their sustainable management. Commercial shark longliners in the marine environment understand habitat compression occurs around oxygen minimum zones and increase their fishing effort (and catch rates) above these areas accordingly [73]. Gorman et al. [74] investigated the depth use of walleye compared to catch rates in their multi-depth gillnet surveys. They found that walleye preferred depth layers higher in the water column in the summer stratified season, which when paired with evidence of avoidance of low DO levels [32], supports our assumptions that walleye avoid the habitat below the thermocline in Hamilton Harbour, and that their depth use is adaptive to interannual variation in environmental conditions. To further support this claim, Larocque et al., (*in review*) found that walleye were shallowest in the Hamilton Harbour water column during the summer than any other season. The abiotic environment in Hamilton Harbour changed on an annual basis, both historically and within this 3-year study, with 2018 having a narrower depth stratum of optimum habitat available to walleye between the surface and the thermocline than the other two years. It is important that fisheries managers understand and account for this variability as sampling catch rates may differ greatly from year to year, regardless of the subpopulation size or characteristics, and could skew the assessment of the subpopulation status.

Understanding environmental drivers of fish movement is also important for fish habitat restoration managers [75]. Habitat availability is often quantified using area and physical/structural habitat attributes [76], and habitat restoration efforts largely focus on physical additions (for example spawning beds, instream structures, riparian planting), or fish passage (dam removal or fish ladders) [77]. This study has highlighted the importance of including abiotic factors like temperature and DO into fish habitat restoration discussions, although they are likely more difficult to restore in shorter time frames than physical habitat alterations. We highlight the value of quantifying habitat in terms of volume, and not just depth as the shape of the lake can dictate what portion of the system below a certain depth is hypoxic. Our findings also illustrate the importance of including multiple abiotic factors. While the relationship between water temperature and DO is often negative and quasi linear, as is expected, this is not always the case; factors such as water chemistry, biotic respiration, and the thermal properties of water (leading to thermal stratification and turnover) all influence the amount of oxygen in the water, complicating the quantification of habitat availability [9].

Many abiotic and biotic factors have been shown to drive fish movement, and light and prey availability are likely important but unquantified drivers of movement [41, 78]. Our analyses were complicated by the confounding factors of hypoxia and optimum habitat, i.e., the best and the absolute worst habitats are only available at a similar time and demonstrated the heterogeneity of the typical three months of summer that often get grouped into one ‘season’ during fish movement studies. Our analysis did not identify the actual temperature and DO that walleye were using, therefore future analyses could investigate walleye movement on a finer scale incorporating depth use and parsing out time periods when hypoxia is present with minimal optimum habitat, or vice versa, which may lead to a stronger relationship between habitat availability and habitat selection. Indeed, the most challenging period for walleye from a DO perspective occurred in the summer of 2018 when hypoxia was prevalent, but optimum habitat was limited. This coincided with a marked reduction in the proportion of tagged individuals that were in the system (as they had left Hamilton Harbour) and may suggest that there are thresholds for when the challenge of reduced DO-based habitat suitability exceed the benefits of habitat compression; increased intraspecific competition may further dictate walleye emigration from the harbour.

Acoustic telemetry has advanced animal movement ecology greatly [79], however there are limitations and assumptions when analyzing telemetry data, particularly with the range or efficiency a receiver can detect a fish, and how the environment can affect this [50, 80]. Brooks et al. [27] determined that walleye used the least space during the summer months and concluded this could be related to hypoxia. More recent work in this study system, however, determined a significant reduction in acoustic telemetry efficiency in the summer due to the thermocline affecting the propagation of sound [52]. Although we used range testing in the coastal areas of Hamilton Harbour and weighted the seasonal home ranges to account for seasonal array efficiency changes in Brooks et al. [27], we felt a more in-depth method was required to account for the strong reduction in performance observed in Wells et al. [52]. This was to ensure we could more accurately answer questions concerning walleye movements changing in response to their environment, as opposed to changes manifested due to our technology failing to detect our animals behaving ‘normally’. Methods to account for such changes in analyses are rare in the fish movement literature. We feel the approach used herein addresses the issue sufficiently and scaled our displacement distances accordingly and as such may hold promise in other systems with similar seasonal changes in detection efficiency.

## Conclusions

Climate induced changes have been documented in hundreds of lakes around the globe, with increasing levels of hypoxia [81] and increasing surface temperatures [82]. Most lentic fish species are unable to migrate too far latitudinally to avoid their changing environment (with lake and watershed boundaries limiting their movements), and likely will start to feel the ‘squeeze’ between a hypoxic hypolimnion and a warmer-than-optimum surface [25], particularly cold- and cool-water species. Results from the current study suggest that this resulting habitat compression leads to reduced movements of top predators like walleye, which could be linked to a greater concentration of prey; however, should future conditions further compress the volume of habitat available to them, they may be forced to seek more thermally stable conditions in the larger and less productive areas of Lake Ontario. It is essential to further our understanding of how apex predator behaviour may change, and the consequent knock-on effects to the rest of the fish community and aquatic ecosystems.

## Supplementary Information

Supplementary file 1.

Supplementary file 2.

Supplementary file 3.

Supplementary file 4.

Supplementary file 5.

Supplementary file 6.

Supplementary file 7.

Supplementary file 8.

Supplementary file 9.

Supplementary file 10.

Supplementary file 11.

Supplementary file 12.

Supplementary file 13.

Supplementary file 14.

Supplementary file 15.

Supplementary file 16.

Supplementary file 17.

Supplementary file 18.

Supplementary file 19.

Supplementary file 20.

## Data Availability

All fish location data has been submitted to the Great Lakes Acoustic Telemetry Observation System’s (https://glatos.glos.us/) database and is available for download with permission from the corresponding author. Fish location detection data is also available in CSV from the corresponding author. Summary data and outputs from the Network Analyses, and all model descriptions, validations and plots are printed in Supplementary Information.
