# Supplementary material for: The influence of thermal and hypoxia induced habitat compression on walleye (Sander vitreus) movements in a temperate lake
Source: Mov Ecol. 2025 Jan 7;13:1. doi: 10.1186/s40462-024-00505-6 (PMC11707865; doi:10.1186/s40462-024-00505-6)

**Supplementary Information**

# **Methods**

*Surgical procedures*

Upon capture, walleye were placed in holding tanks filled with ambient lake water prior to surgical procedures. Fish were anaesthetized using either a Portable Electroanesthesia System (Smith-Root; 81, 82), e-gloves (Smith-Root; 82) or using the boat’s e-fishing electrodes. All methods work by placing the fish in a state of electroanesthesia, which is commonly used during fish surgeries (82). Individuals were placed in a padded trough, oriented ventrally, and ambient lake water was poured or pumped into the trough to cover both the head and gills to maintain normal respiration during the surgeries. Transmitters fitted with pressure sensors to determine depth (InnovaSea™ V13P-1x-069k-1-0034m, 13mm diameter, dry mass 11 g, battery life 1386 days, depth accuracy ± 1.7 m, resolution 0.15 m, depth information used to determine if fish were dead or had expelled their tag) were inserted into the body cavity through a 2-3 cm mid-ventral incision; depth data were only used to assess individual survival. The acoustic transmitters were programmed with a random delay range of 120-270 seconds to reduce transmitter collisions. Incisions were closed with 2-3 interrupted sutures (3-0 polydioxanone-II violet monofilament; Ethicon USA), tied with a double surgeon’s knot. All surgical equipment and transmitters were cleaned with 10% povidone-iodine solution (Betadine®, USA) between each surgery. We measured the fork lengths (mm) of each fish and placed them into a recovery live well containing fresh, recirculating lake water. To ensure full recovery of fish prior to release, fish were tested for sufficient equilibrium, body flex, tail clamp, and eye movement (83). Fish handling and surgical procedures were approved and followed a Canadian Council on Animal Care protocol administered by Carleton University (#110723).

## *Data filtering- time lags*

Data were filtered based on two different types of time intervals, the first was to remove any single, possibly spurious detections with over 3600 s between them, the second was to remove any detections less than the minimum tag pinging interval (here 120 s) to remove duplicated detections on overlapping receivers. We calculated two time-intervals for each fish detection. Firstly, the number of seconds since that transmitter was last detected on the same receiver, and secondly, the number of seconds since that transmitter was last detected on any receiver. The first interval was calculated using the ‘min_lag’ function in the GLATOS R package (https://gitlab.oceantrack.org/GreatLakes/glatos). The second was calculated in R using the time difference between each individual transmitter’s row of data. The objective of the min_lag time interval is to be able to filter out any potential false-positive detections (everything over 3600 s was presumed false positive and was removed). The objective of the time difference interval was to remove duplicate detections from a single transmission in areas with a high degree of receiver coverage overlap. The minimum interval between subsequent transmissions of a tag is 120 s, therefore any time difference less than 120 s was removed as it was presumed to be a duplication. This reduces the risk of artificially inflating the ‘presence’ of a fish in an area with a high degree of overlap in acoustic range.

## *Seasonal adjustment factor calculations.*

For each receiver combination, we calculated an adjustment factor based on the combined mean distance of acoustic coverage for both receivers within the least-cost path distance between those receivers, for both stratified and isothermal conditions:

Proportion of non-coverage between receiver A and B during stratified month = (Distance between A and B – (receiver A stratified mean range + receiver B stratified mean range))/ Distance between A and B

We then calculated the maximum adjustment factor for any receiver pairing in the harbour during that time period and rescaled all other adjustment factors to a value between 0 and 1, based on the maximum:

Adjustment factor for receiver A and B= Proportion of non-coverage for receiver A and B during stratified period/Maximum proportion of non-coverage for all receiver combinations during stratified period.

Receivers that overlapped with their mean detection ranges received an adjustment factor of 0 (i.e., when the total least-cost path distance between receiver A and receiver B was either the exact same as the sum of the two mean detection ranges, or less, signifying overlap). This was to account for the fact that receivers were positioned more densely in some areas and reductions in detection range would matter much less here than receivers that were spread further apart.

*Network Analysis*

Filtered data (based on time intervals, see ‘Data processing’ section) were converted to local time and subset to the 21 receivers for our nodes. Network Analysis was conducted if detected on at least two nodes, and the walleye was detected on at least 75% of the days in that month (to minimize bias from walleye departing the study system midway through a month). To account for spatial bias in the array design (i.e., a movement between two nodes is more likely than another pair because the nodes are closer), each network was tested for non-random associations with a link rearrangement and bootstrap approach (53-55. The observed movements were shuffled between receivers, and the node degree metrics from the original network were used to make 10,000 random graphs with the same degree distribution (53-55). Global transitivity is the probability that the adjacent vertices of a vertex are connected, also known as the clustering coefficient, and is the ratio of the count of triangles and connected triples in the graph (84, 85). This metric was calculated for the walleye network and then compared to that of the random networks and if it was outside +/- 2*SD from the mean, it was deemed to be a non-random graph, i.e., the network was representative of that fish’s behaviour as opposed to a signature of the array’s spatial formation.

*References*

81. Reid CH, Vandergoot CS, Midwood JD, Stevens ED, Bowker J, Cooke SJ. On the Electroimmobilization of Fishes for Research and Practice: Opportunities, Challenges, and Research Needs. Fisheries. 2019;44(12):576–85.

82. Reid CH, Faust MD, Raby GD, Brenden TO, Cooke SJ, Vandergoot CS. Postrelease Survival and Migration Behavior of Adult Walleye Following Intracoelomic Transmitter Implantation Using Two Methods of Electro-Immobilization. Trans Am Fish Soc. 2022;151(1):100–11.

83. Raby GD, Donaldson MR, Hinch SG, Patterson DA, Lotto AG, Robichaud D, et al. Validation of reflex indicators for measuring vitality and predicting the delayed mortality of wild coho salmon bycatch released from fishing gears. J Appl Ecol. 2012;49(1):90–8.

84. Barrat A, Barthélemy M, Pastor-Satorras R, Vespignani A. The architecture of complex weighted networks. Proc Natl Acad Sci. 2004 Mar 16;101(11):3747–52.

85. Bastille-Rousseau G, Douglas-Hamilton I, Blake S, Northrup JM, Wittemyer G. Applying network theory to animal movements to identify properties of landscape space use. Ecol Appl. 2018;28(3):854–64.

*Figure Captions*

Figure 1. Hypsographic curve obtained from the Digital Elevation Model of Hamilton Harbour (excluding Cootes Paradise). Digital Elevation Model of Hamilton Harbour, excluding Cootes Paradise as walleye have not been documented to use the marsh area.

Figure 2. Map of the array with the mean detection range during the stratified (orange) and isothermal (blue) periods (data obtained from Wells et al. 2021).

Figure 3. Least cost path calculations (m) between each pair of receivers in the 21-station array. Although lines show paths over land, the distances were calculated ‘as the fish swims’, i.e., around the land border.

Figure 4. Network for walleye #83 in a) September 2016 and b) June 2016. Thickness of connecting lines indicates the number of movements between those two nodes (receiver stations). Node colours indicate geographic region of the Harbour.

Figure 5. Categorized abiotic conditions per 1 m depth for each day of the year for every year data were available (one panel per decade). Black shading is hypoxic (<3 mg/L), green represents physiologically optimal for walleye (Sander vitreus; 18 – 23 °C, > 5 mg/L), and white is suitable (all temperatures, > 3 mg/L).

GAMM analyses- model outputs and model check

# Model 1

# Displacement distance will be influenced by the volume of Suitable habitat and the size of the individual walleye.

# gam(distance_km ~ s(Suitable)+ s(Est_FL) + s(wall_ID, bs=‘re’) + s(Year, bs=‘re’), data=df2, method=‘REML’, family = gaussian(link = “log”))

##
## Family: gaussian
## Link function: log
##
## Formula:
## distance_km ~ s(Suitable) + s(Est_FL) + s(wall_ID, bs = "re") +
## s(Year, bs = "re")
##
## Parametric coefficients:
## Estimate Std. Error t value Pr(>|t|)
## (Intercept) 7.5579 0.2459 30.73 <2e-16 ***
## ---
## Signif. codes: 0 '***' 0.001 '**' 0.01 '*' 0.05 '.' 0.1 ' ' 1
##
## Approximate significance of smooth terms:
## edf Ref.df F p-value
## s(Suitable) 3.496 4.133 1.507 0.2281
## s(Est_FL) 1.001 1.002 0.131 0.7192
## s(wall_ID) 17.018 31.000 2.335 4.66e-07 ***
## s(Year) 1.678 2.000 8.545 0.0188 *
## ---
## Signif. codes: 0 '***' 0.001 '**' 0.01 '*' 0.05 '.' 0.1 ' ' 1
##
## R-sq.(adj) = 0.388 Deviance explained = 48.6%
## -REML = 1255.2 Scale est. = 2.5344e+06 n = 140


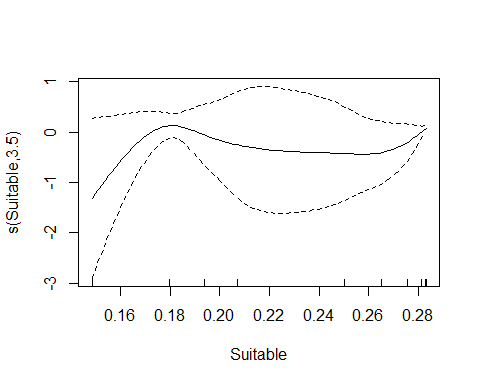

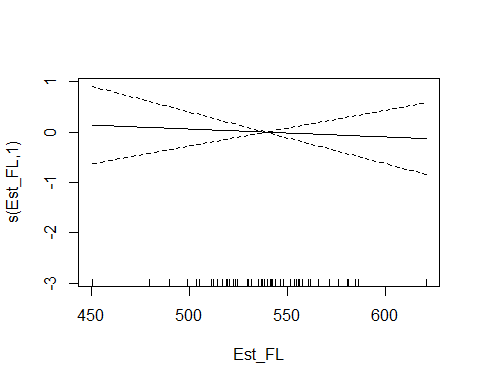

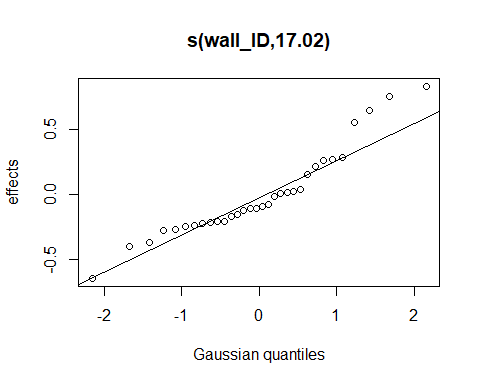

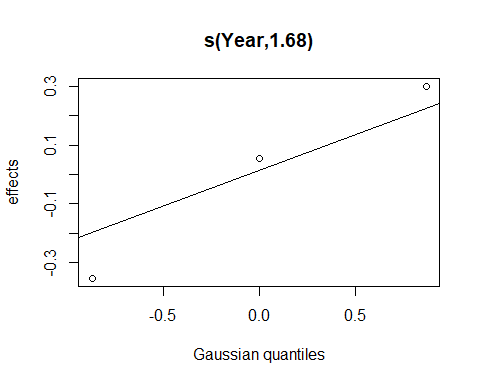

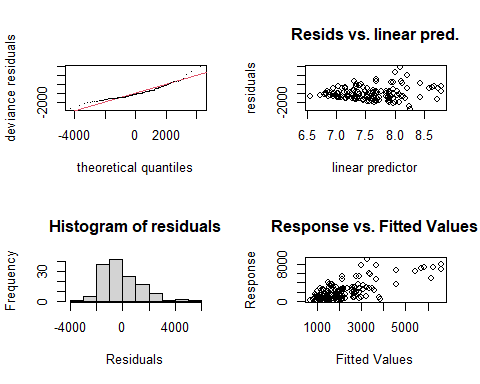


##
## Method: REML Optimizer: outer newton
## full convergence after 9 iterations.
## Gradient range [-0.0004841417,0.0002198162]
## (score 1255.191 & scale 2534390).
## Hessian positive definite, eigenvalue range [0.0004837533,69.55554].
## Model rank = 54 / 54
##
## Basis dimension (k) checking results. Low p-value (k-index<1) may
## indicate that k is too low, especially if edf is close to k'.
##
## k' edf k-index p-value
## s(Suitable) 9.00 3.50 0.84 0.02 *
## s(Est_FL) 9.00 1.00 1.03 0.60
## s(wall_ID) 32.00 17.02 NA NA
## s(Year) 3.00 1.68 NA NA
## ---
## Signif. codes: 0 '***' 0.001 '**' 0.01 '*' 0.05 '.' 0.1 ' ' 1


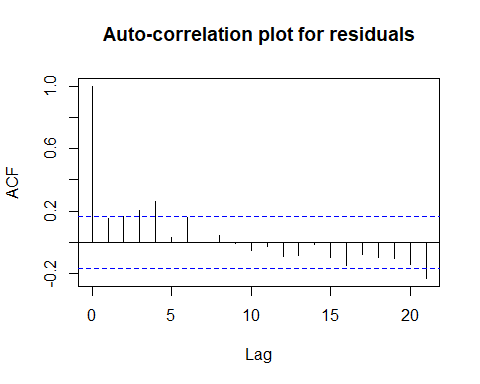

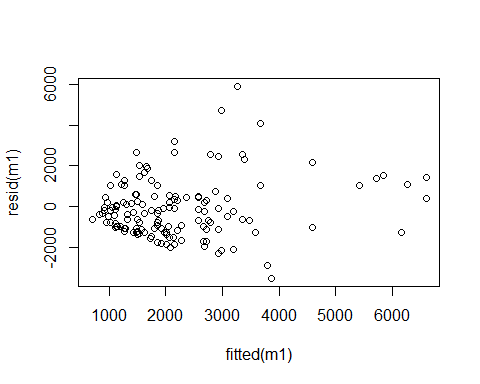


## para s(Suitable) s(Est_FL) s(wall_ID) s(Year)
## worst 1 0.6076232 0.9998650 1.0000000 1.0000000
## observed 1 0.2321544 0.9962618 0.3330843 0.6091015
## estimate 1 0.3299513 0.9519434 0.3141844 0.8421940

## $worst
## para s(Suitable) s(Est_FL) s(wall_ID) s(Year)
## para 1.000000e+00 1.121480e-16 3.098092e-23 1.0000000 1.0000000
## s(Suitable) 1.149075e-16 1.000000e+00 1.590869e-01 0.4916594 0.2724698
## s(Est_FL) 3.098838e-23 1.590869e-01 1.000000e+00 0.9998095 0.4381112
## s(wall_ID) 1.000000e+00 4.916594e-01 9.998095e-01 1.0000000 1.0000000
## s(Year) 1.000000e+00 2.724698e-01 4.381112e-01 1.0000000 1.0000000
##
## $observed
## para s(Suitable) s(Est_FL) s(wall_ID) s(Year)
## para 1.000000e+00 4.183976e-26 3.139278e-32 0.004163244 0.03851251
## s(Suitable) 1.149075e-16 1.000000e+00 9.461168e-02 0.082280321 0.21124688
## s(Est_FL) 3.098838e-23 2.194378e-02 1.000000e+00 0.135368826 0.11115925
## s(wall_ID) 1.000000e+00 1.839229e-01 9.567832e-01 1.000000000 0.30928554
## s(Year) 1.000000e+00 8.969821e-03 3.398353e-01 0.155775865 1.00000000
##
## $estimate
## para s(Suitable) s(Est_FL) s(wall_ID) s(Year)
## para 1.000000e+00 1.339853e-30 3.125165e-25 0.03877551 0.3564286
## s(Suitable) 1.149075e-16 1.000000e+00 6.548904e-02 0.05317204 0.1428013
## s(Est_FL) 3.098838e-23 4.155779e-02 1.000000e+00 0.19441979 0.1828159
## s(wall_ID) 1.000000e+00 1.668854e-01 9.233491e-01 1.00000000 0.6566043
## s(Year) 1.000000e+00 4.982783e-02 1.883877e-01 0.07009849 1.0000000


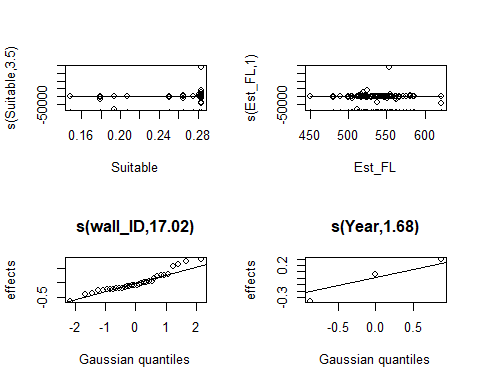

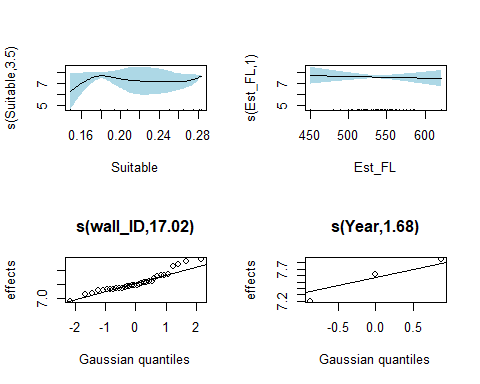


# Model 2

# Displacement distance will be influenced by the volume of Optimum.

# gam(distance_km ~ s(Optimum) + s(wall_ID, bs=‘re’) + s(Year, bs=‘re’), data=df2, method=‘REML’, family = gaussian(link = “log”))

##
## Family: gaussian
## Link function: log
##
## Formula:
## distance_km ~ s(Optimum) + s(wall_ID, bs = "re") + s(Year, bs = "re")
##
## Parametric coefficients:
## Estimate Std. Error t value Pr(>|t|)
## (Intercept) 7.4970 0.2418 31.01 <2e-16 ***
## ---
## Signif. codes: 0 '***' 0.001 '**' 0.01 '*' 0.05 '.' 0.1 ' ' 1
##
## Approximate significance of smooth terms:
## edf Ref.df F p-value
## s(Optimum) 3.676 4.465 4.635 0.00165 **
## s(wall_ID) 16.869 31.000 5.837 < 2e-16 ***
## s(Year) 1.775 2.000 8.809 0.03644 *
## ---
## Signif. codes: 0 '***' 0.001 '**' 0.01 '*' 0.05 '.' 0.1 ' ' 1
##
## R-sq.(adj) = 0.44 Deviance explained = 52.6%
## -REML = 1248.1 Scale est. = 2.3204e+06 n = 140


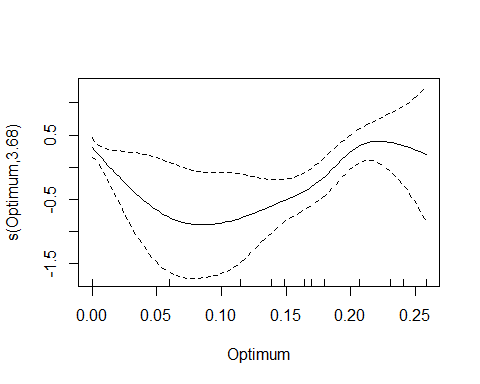

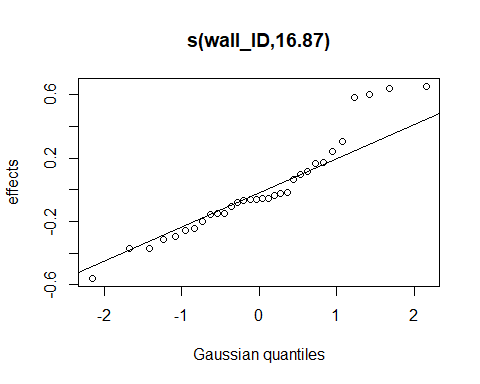

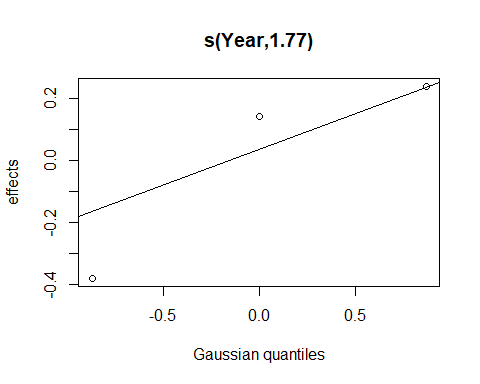

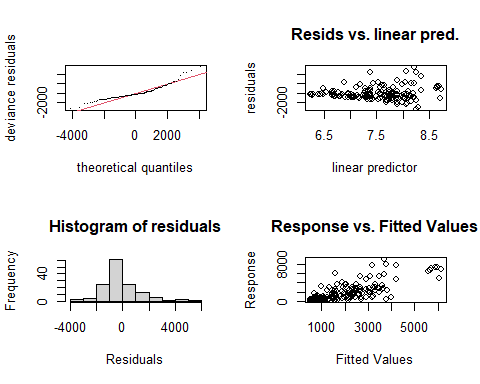


##
## Method: REML Optimizer: outer newton
## full convergence after 6 iterations.
## Gradient range [-1.128272e-05,5.796202e-06]
## (score 1248.103 & scale 2320405).
## Hessian positive definite, eigenvalue range [0.4743741,70.06766].
## Model rank = 45 / 45
##
## Basis dimension (k) checking results. Low p-value (k-index<1) may
## indicate that k is too low, especially if edf is close to k'.
##
## k' edf k-index p-value
## s(Optimum) 9.00 3.68 0.88 0.065 .
## s(wall_ID) 32.00 16.87 NA NA
## s(Year) 3.00 1.77 NA NA
## ---
## Signif. codes: 0 '***' 0.001 '**' 0.01 '*' 0.05 '.' 0.1 ' ' 1


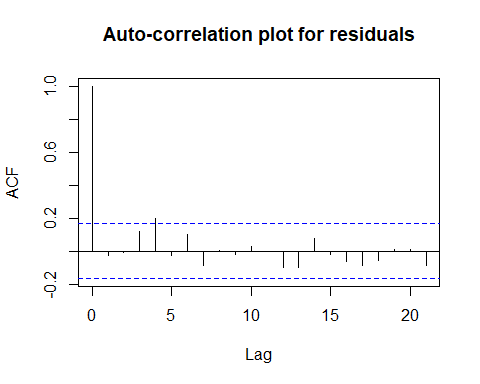

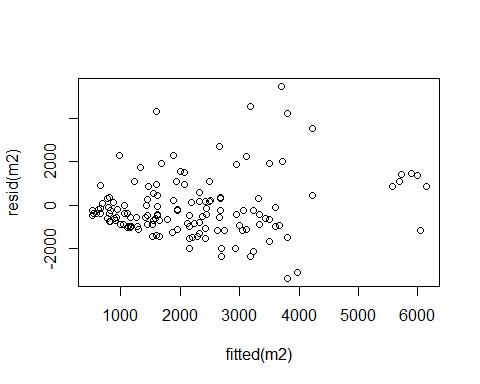


## para s(Optimum) s(wall_ID) s(Year)
## worst 1 0.6802050 1.0000000 1.0000000
## observed 1 0.2210087 0.2310548 0.5837902
## estimate 1 0.3153724 0.1241372 0.7137570

## $worst
## para s(Optimum) s(wall_ID) s(Year)
## para 1.00000e+00 2.508257e-22 1.0000000 1.0000000
## s(Optimum) 2.50594e-22 1.000000e+00 0.6222415 0.3589353
## s(wall_ID) 1.00000e+00 6.222415e-01 1.0000000 1.0000000
## s(Year) 1.00000e+00 3.589353e-01 1.0000000 1.0000000
##
## $observed
## para s(Optimum) s(wall_ID) s(Year)
## para 1.00000e+00 3.179941e-28 0.004075192 0.05827497
## s(Optimum) 2.50594e-22 1.000000e+00 0.059106308 0.32441369
## s(wall_ID) 1.00000e+00 1.875800e-01 1.000000000 0.44207491
## s(Year) 1.00000e+00 3.681999e-02 0.191511519 1.00000000
##
## $estimate
## para s(Optimum) s(wall_ID) s(Year)
## para 1.00000e+00 2.522416e-25 0.03877551 0.3564286
## s(Optimum) 2.50594e-22 1.000000e+00 0.05153092 0.1625886
## s(wall_ID) 1.00000e+00 2.672344e-01 1.00000000 0.6566043
## s(Year) 1.00000e+00 1.234730e-02 0.07009849 1.0000000


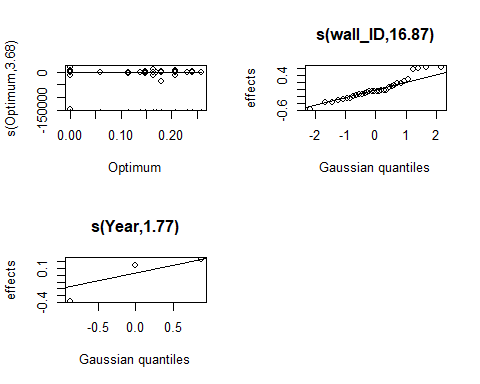

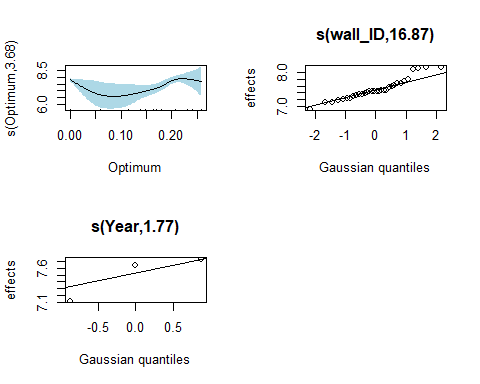


# Model 3

# Although Suitable habitat is reduced during the summer, a portion of the remaining habitat is of higher quality. We hypothesize that the amount of Optimum habitat will also influence displacement distances of walleye. To determine this, we subset the dataset to only include the months that contained hypoxia and tested both habitat categories.

# m3<-gam(distance_km ~ s(Suitable) + s(Year, bs=‘re’) + s(wall_ID, bs=‘re’), data = df3, method=‘REML’, family = gaussian(link = “log”))

##
## Family: gaussian
## Link function: log
##
## Formula:
## distance_km ~ s(Suitable) + s(Year, bs = "re") + s(wall_ID, bs = "re")
##
## Parametric coefficients:
## Estimate Std. Error t value Pr(>|t|)
## (Intercept) 6.9796 0.4449 15.69 <2e-16 ***
## ---
## Signif. codes: 0 '***' 0.001 '**' 0.01 '*' 0.05 '.' 0.1 ' ' 1
##
## Approximate significance of smooth terms:
## edf Ref.df F p-value
## s(Suitable) 4.912 5.294 5.944 0.000242 ***
## s(Year) 1.642 2.000 572.404 0.000830 ***
## s(wall_ID) 19.106 29.000 20.794 9.14e-05 ***
## ---
## Signif. codes: 0 '***' 0.001 '**' 0.01 '*' 0.05 '.' 0.1 ' ' 1
##
## R-sq.(adj) = 0.803 Deviance explained = 87.4%
## -REML = 623.84 Scale est. = 7.7132e+05 n = 72


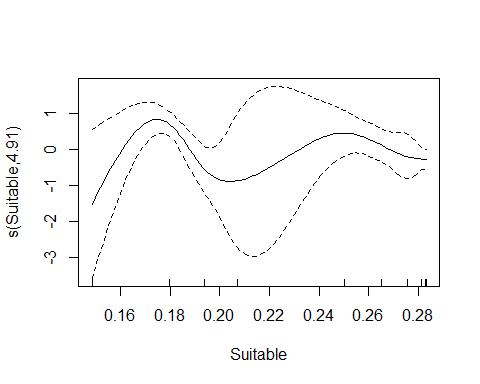

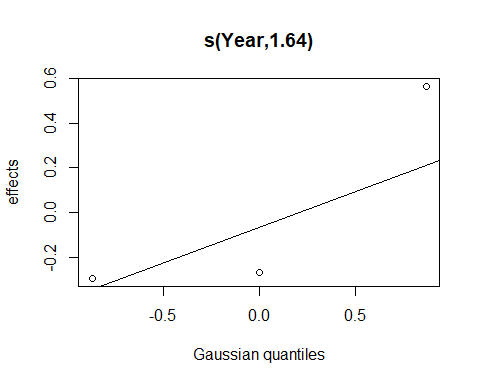

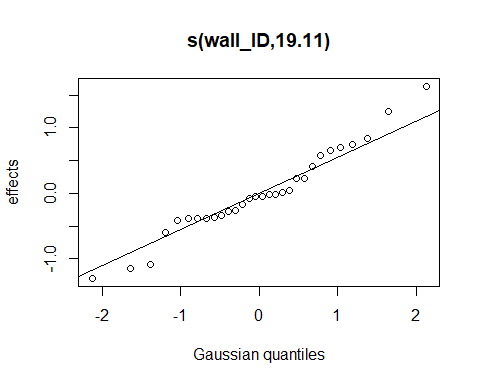

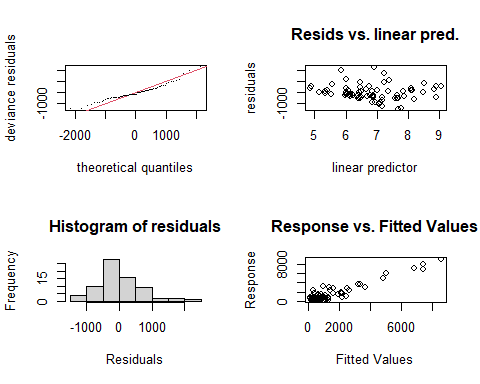


##
## Method: REML Optimizer: outer newton
## full convergence after 6 iterations.
## Gradient range [-0.0001957419,-5.363203e-06]
## (score 623.8397 & scale 771322.3).
## Hessian positive definite, eigenvalue range [0.6061312,37.21415].
## Model rank = 43 / 43
##
## Basis dimension (k) checking results. Low p-value (k-index<1) may
## indicate that k is too low, especially if edf is close to k'.
##
## k' edf k-index p-value
## s(Suitable) 9.00 4.91 0.98 0.40
## s(Year) 3.00 1.64 NA NA
## s(wall_ID) 30.00 19.11 NA NA

## null device
## 1

## para s(Suitable) s(Year) s(wall_ID)
## worst 1 0.8163107 1.0000000 1.0000000
## observed 1 0.5073692 0.6467026 0.1119030
## estimate 1 0.6288934 0.8601209 0.1902697

## $worst
## para s(Suitable) s(Year) s(wall_ID)
## para 1.000000e+00 1.645374e-17 1.0000000 1.0000000
## s(Suitable) 1.820593e-17 1.000000e+00 0.5259137 0.6738056
## s(Year) 1.000000e+00 5.259137e-01 1.0000000 1.0000000
## s(wall_ID) 1.000000e+00 6.738056e-01 1.0000000 1.0000000
##
## $observed
## para s(Suitable) s(Year) s(wall_ID)
## para 1.000000e+00 5.147138e-27 0.01296054 0.01272519
## s(Suitable) 1.820593e-17 1.000000e+00 0.35171896 0.04217504
## s(Year) 1.000000e+00 3.925032e-02 1.00000000 0.08016058
## s(wall_ID) 1.000000e+00 2.186877e-01 0.50748161 1.00000000
##
## $estimate
## para s(Suitable) s(Year) s(wall_ID)
## para 1.000000e+00 2.921443e-31 0.3846451 0.04552469
## s(Suitable) 1.820593e-17 1.000000e+00 0.2574976 0.10387855
## s(Year) 1.000000e+00 1.672381e-01 1.0000000 0.09182933
## s(wall_ID) 1.000000e+00 4.235661e-01 0.8032407 1.00000000

# Model 4

# Optimum habitat availability will influence walleye displacement distances.

# m4<-gam(distance_km ~ s(Optimum) + s(Year, bs=‘re’) + s(wall_ID, bs=‘re’), data = df3, method=‘REML’, family = gaussian(link = “log”))

##
## Family: gaussian
## Link function: log
##
## Formula:
## distance_km ~ s(Optimum) + s(Year, bs = "re") + s(wall_ID, bs = "re")
##
## Parametric coefficients:
## Estimate Std. Error t value Pr(>|t|)
## (Intercept) 6.8592 0.5092 13.47 <2e-16 ***
## ---
## Signif. codes: 0 '***' 0.001 '**' 0.01 '*' 0.05 '.' 0.1 ' ' 1
##
## Approximate significance of smooth terms:
## edf Ref.df F p-value
## s(Optimum) 1.002 1.003 27.28 3.53e-06 ***
## s(Year) 1.749 2.000 505.05 0.00805 **
## s(wall_ID) 17.401 29.000 45.93 0.00495 **
## ---
## Signif. codes: 0 '***' 0.001 '**' 0.01 '*' 0.05 '.' 0.1 ' ' 1
##
## R-sq.(adj) = 0.721 Deviance explained = 79.9%
## -REML = 627.28 Scale est. = 1.0925e+06 n = 72


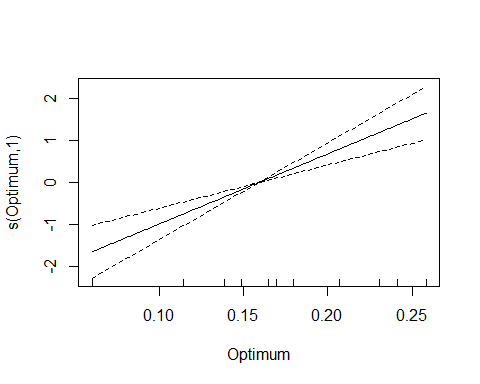

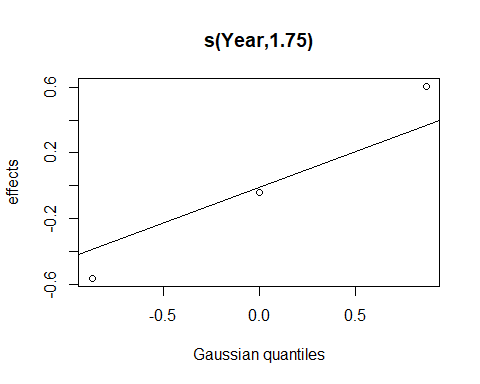

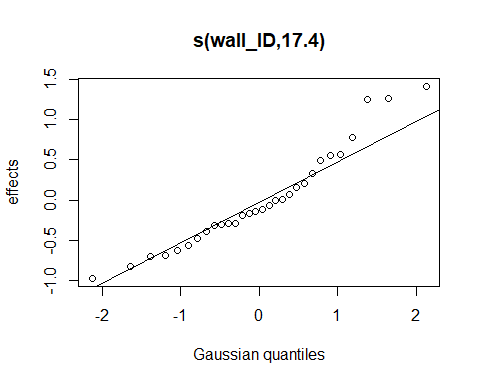

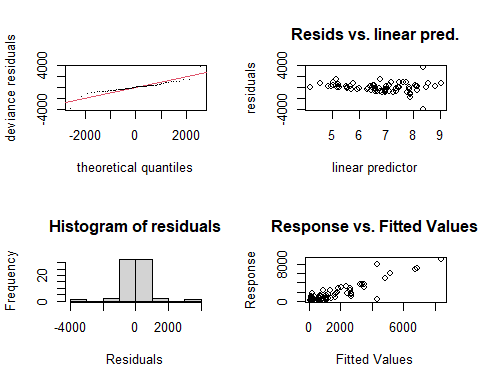


##
## Method: REML Optimizer: outer newton
## full convergence after 9 iterations.
## Gradient range [-0.0002219102,0.0006489329]
## (score 627.2805 & scale 1092514).
## Hessian positive definite, eigenvalue range [0.0001684593,36.95934].
## Model rank = 43 / 43
##
## Basis dimension (k) checking results. Low p-value (k-index<1) may
## indicate that k is too low, especially if edf is close to k'.
##
## k' edf k-index p-value
## s(Optimum) 9.00 1.00 1 0.48
## s(Year) 3.00 1.75 NA NA
## s(wall_ID) 30.00 17.40 NA NA

## null device
## 1

## para s(Optimum) s(Year) s(wall_ID)
## worst 1 0.9126438 1.0000000 1.0000000
## observed 1 0.6321830 0.8270786 0.1635268
## estimate 1 0.5990895 0.9189400 0.1901393

## $worst
## para s(Optimum) s(Year) s(wall_ID)
## para 1.000000e+00 2.350619e-26 1.0000000 1.0000000
## s(Optimum) 2.368178e-26 1.000000e+00 0.7627882 0.7546963
## s(Year) 1.000000e+00 7.627882e-01 1.0000000 1.0000000
## s(wall_ID) 1.000000e+00 7.546963e-01 1.0000000 1.0000000
##
## $observed
## para s(Optimum) s(Year) s(wall_ID)
## para 1.000000e+00 3.874410e-34 0.005227487 0.02298401
## s(Optimum) 2.368178e-26 1.000000e+00 0.714114367 0.09373453
## s(Year) 1.000000e+00 6.051306e-02 1.000000000 0.06587288
## s(wall_ID) 1.000000e+00 4.493470e-01 0.431494005 1.00000000
##
## $estimate
## para s(Optimum) s(Year) s(wall_ID)
## para 1.000000e+00 1.144658e-28 0.3846451 0.04552469
## s(Optimum) 2.368178e-26 1.000000e+00 0.3633295 0.10542438
## s(Year) 1.000000e+00 6.788212e-02 1.0000000 0.09182933
## s(wall_ID) 1.000000e+00 4.530550e-01 0.8032407 1.00000000

# Model 5

# Both categories of habitat significantly influence displacement distances of walleye, and the amount of each varies throughout the summer. We would predict that Optimum habitat is a stronger predictor of walleye displacement.

# m5<-gam(distance_km ~ s(Optimum) + s(Suitable)+ s(Year, bs=‘re’) + s(wall_ID, bs=‘re’), data = df3, method=‘REML’, family = gaussian(link = “log”))

##
## Family: gaussian
## Link function: log
##
## Formula:
## distance_km ~ s(Optimum) + s(Suitable) + s(Year, bs = "re") +
## s(wall_ID, bs = "re")
##
## Parametric coefficients:
## Estimate Std. Error t value Pr(>|t|)
## (Intercept) 6.6555 0.5404 12.31 1.98e-15 ***
## ---
## Signif. codes: 0 '***' 0.001 '**' 0.01 '*' 0.05 '.' 0.1 ' ' 1
##
## Approximate significance of smooth terms:
## edf Ref.df F p-value
## s(Optimum) 3.585 4.127 5.204 0.00167 **
## s(Suitable) 3.488 3.923 1.816 0.13918
## s(Year) 1.759 2.000 1387.398 0.00475 **
## s(wall_ID) 20.759 29.000 13.239 9.27e-05 ***
## ---
## Signif. codes: 0 '***' 0.001 '**' 0.01 '*' 0.05 '.' 0.1 ' ' 1
##
## R-sq.(adj) = 0.863 Deviance explained = 92%
## -REML = 617.74 Scale est. = 5.3385e+05 n = 72


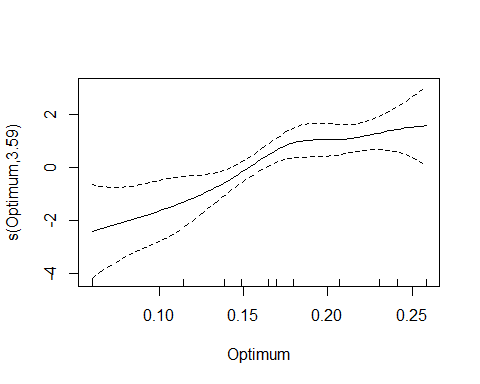

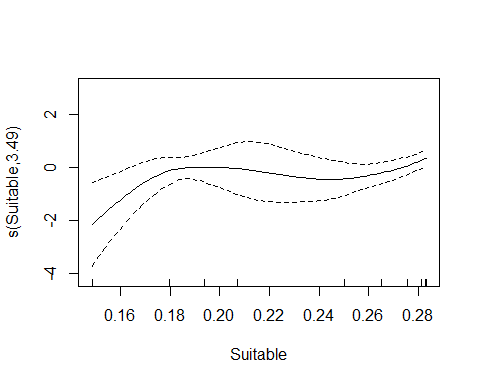

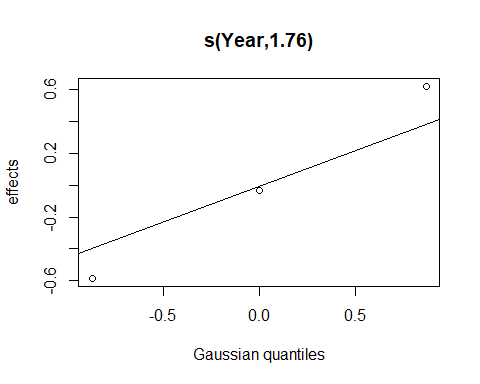

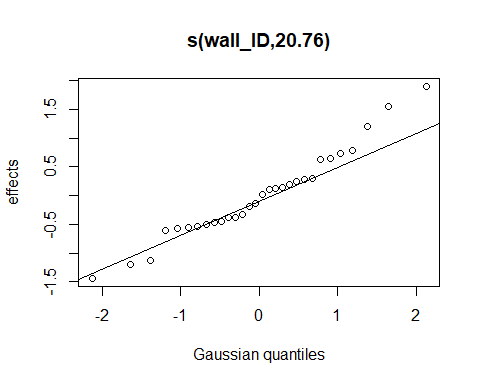

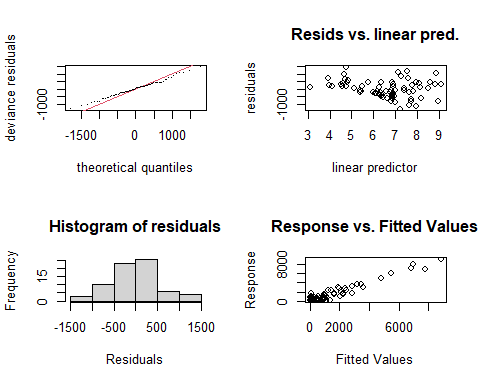


##
## Method: REML Optimizer: outer newton
## full convergence after 9 iterations.
## Gradient range [-6.825359e-05,3.066357e-05]
## (score 617.7417 & scale 533851).
## Hessian positive definite, eigenvalue range [0.3355113,36.86922].
## Model rank = 52 / 52
##
## Basis dimension (k) checking results. Low p-value (k-index<1) may
## indicate that k is too low, especially if edf is close to k'.
##
## k' edf k-index p-value
## s(Optimum) 9.00 3.59 1.00 0.43
## s(Suitable) 9.00 3.49 1.02 0.52
## s(Year) 3.00 1.76 NA NA
## s(wall_ID) 30.00 20.76 NA NA

## null device
## 1

## para s(Optimum) s(Suitable) s(Year) s(wall_ID)
## worst 1 1.0000000 1.0000000 1.0000000 1.0000000
## observed 1 0.9435161 0.6971286 0.9772341 0.2583230
## estimate 1 0.9311875 0.9936074 0.9810398 0.3012498

## $worst
## para s(Optimum) s(Suitable) s(Year) s(wall_ID)
## para 1.000000e+00 2.350619e-26 1.645374e-17 1.0000000 1.0000000
## s(Optimum) 2.368178e-26 1.000000e+00 1.000000e+00 0.7627882 0.7546963
## s(Suitable) 2.370277e-17 1.000000e+00 1.000000e+00 0.5259137 0.6738056
## s(Year) 1.000000e+00 7.627882e-01 5.259137e-01 1.0000000 1.0000000
## s(wall_ID) 1.000000e+00 7.546963e-01 6.738056e-01 1.0000000 1.0000000
##
## $observed
## para s(Optimum) s(Suitable) s(Year) s(wall_ID)
## para 1.000000e+00 4.473241e-32 3.371294e-27 0.005853117 0.01786037
## s(Optimum) 2.368178e-26 1.000000e+00 5.692385e-01 0.715917093 0.09647641
## s(Suitable) 2.370277e-17 7.917502e-01 1.000000e+00 0.433514952 0.04846091
## s(Year) 1.000000e+00 8.183516e-02 4.726382e-02 1.000000000 0.06668919
## s(wall_ID) 1.000000e+00 4.349769e-01 3.950801e-01 0.432521030 1.00000000
##
## $estimate
## para s(Optimum) s(Suitable) s(Year) s(wall_ID)
## para 1.000000e+00 1.144658e-28 2.921443e-31 0.3846451 0.04552469
## s(Optimum) 2.368178e-26 1.000000e+00 9.844588e-01 0.3633295 0.10542438
## s(Suitable) 2.370277e-17 7.657720e-01 1.000000e+00 0.2574976 0.10387855
## s(Year) 1.000000e+00 6.788212e-02 1.672381e-01 1.0000000 0.09182933
## s(wall_ID) 1.000000e+00 4.530550e-01 4.235661e-01 0.8032407 1.00000000

# Concurvity values show some correlation between the amount of Suitable and Optimum habitat, however plots show a weak linear, and an interesting non-linear relationship.

## `geom_smooth()` using formula 'y ~ x'


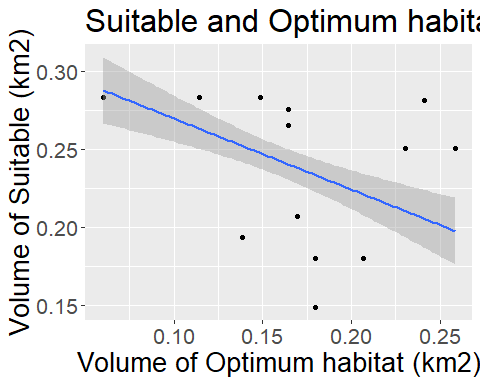


## `geom_smooth()` using formula 'y ~ x'


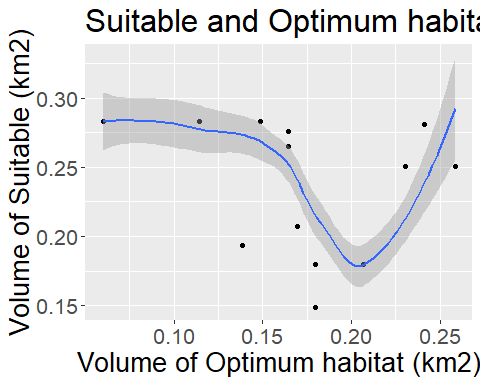

Supplement: Supplementary file 1 [file 40462_2024_505_MOESM1_ESM.docx]
