# Supplementary material for: The influence of thermal and hypoxia induced habitat compression on walleye (Sander vitreus) movements in a temperate lake
Source: Mov Ecol. 2025 Jan 7;13:1. doi: 10.1186/s40462-024-00505-6 (PMC11707865; doi:10.1186/s40462-024-00505-6)
Supplement: Supplementary file 2 [file 40462_2024_505_MOESM2_ESM.docx]

Table 1. Walleye (S*ander vitreus*) caught and tagged in Hamilton Harbour during the study between 2015 and 2018, including projected fork lengths based on age and growth data for each year of the study.

| **Walleye ID** | **Total Length (mm)** | **Release Date** | **Fork Length (mm)** | **Estimated Age** | **FL (2015)** | **FL (2016)** | **FL (2017)** | **FL (2018)** |
| --- | --- | --- | --- | --- | --- | --- | --- | --- |
| 15755 | 490 | 8/12/2015 | 466 | 3 | 466 | 499 | 523 | 541 |
| 15760 | 512 | 8/13/2015 | 487 | 4 | 487 | 511 | 528 | 541 |
| 15766 | 430 | 8/13/2015 | 409 | 2 | 409 | 450 | 482 | 505 |
| 15769 | 513 | 8/13/2015 | 488 | 4 | 488 | 512 | 529 | 542 |
| 15759 | 471 | 8/13/2015 | 448 | 3 | 448 | 480 | 503 | 520 |
| 15771 | 562 | 10/20/2015 | 535 | 6 | 535 | 548 | 557 | 564 |
| 15764 | 570 | 10/20/2015 | 542 | 6 | 542 | 555 | 565 | 572 |
| 79 | 520 | 10/20/2015 | 495 | 4 | 495 | 519 | 537 | 550 |
| 83 | 515 | 10/20/2015 | 490 | 4 | 490 | 514 | 532 | 544 |
| 15774 | 525 | 10/20/2015 | 500 | 4 | 500 | 524 | 542 | 555 |
| 15765 | 521 | 10/20/2015 | 496 | 4 | 496 | 520 | 538 | 551 |
| 15772 | 555 | 10/20/2015 | 528 | 5 | 528 | 546 | 559 | 569 |
| 15763 | 506 | 10/20/2015 | 481 | 4 | 481 | 505 | 522 | 535 |
| 18972 | NA | 6/16/2016 | 490 | 4 |  | 490 | 506 | 519 |
| 18969 | NA | 6/21/2016 | 500 | 4 |  | 500 | 517 | 529 |
| 18973 | 545 | 6/29/2016 | 519 | 5 |  | 519 | 536 | 549 |
| 18965 | 590 | 6/30/2016 | 561 | 7 |  | 561 | 571 | 578 |
| 18966 | 557 | 6/30/2016 | 530 | 6 |  | 530 | 539 | 546 |
| 18967 | 550 | 6/30/2016 | 523 | 5 |  | 523 | 541 | 554 |
| 16053 | 610 | 4/13/2017 | 581 | 9 |  |  | 581 | 586 |
| 16063 | 605 | 4/13/2017 | 576 | 9 |  |  | 576 | 581 |
| 16051 | 584 | 4/18/2017 | 556 | 7 |  |  | 556 | 565 |
| 16052 | 579 | 4/18/2017 | 551 | 7 |  |  | 551 | 561 |
| 16061 | 562 | 4/18/2017 | 535 | 6 |  |  | 535 | 541 |
| 16062 | 579 | 4/18/2017 | 551 | 7 |  |  | 551 | 561 |
| 16055 | 574 | 4/19/2017 | 546 | 6 |  |  | 546 | 553 |
| 16056 | 610 | 4/19/2017 | 581 | 10 |  |  | 581 | 584 |
| 16057 | 652 | 4/19/2017 | 621 | 20 |  |  | 621 | 621 |
| 16058 | 556 | 4/19/2017 | 529 | 5 |  |  | 529 | 542 |
| 16059 | 564 | 4/19/2017 | 537 | 6 |  |  | 537 | 543 |
| 14519 | 590 | 5/23/2018 | 561 | 7 |  |  |  | 561 |
| 14516 | 550 | 5/24/2018 | 523 | 5 |  |  |  | 523 |
