# Supplementary material for: The influence of thermal and hypoxia induced habitat compression on walleye (Sander vitreus) movements in a temperate lake
Source: Mov Ecol. 2025 Jan 7;13:1. doi: 10.1186/s40462-024-00505-6 (PMC11707865; doi:10.1186/s40462-024-00505-6)
Supplement: Supplementary file 3 [file 40462_2024_505_MOESM3_ESM.docx]

Table 2. Volume at depth for Hamilton Harbour.

| **Depth** | **Volume (m³)** |
| --- | --- |
| 0 | 20859625 |
| -1 | 20128575 |
| -2 | 19007175 |
| -3 | 18531025 |
| -4 | 18160350 |
| -5 | 17773500 |
| -6 | 17296350 |
| -7 | 16752900 |
| -8 | 16073300 |
| -9 | 15269200 |
| -10 | 13988700 |
| -11 | 13104800 |
| -12 | 12281250 |
| -13 | 11474875 |
| -14 | 10444750 |
| -15 | 9268750 |
| -16 | 8061450 |
| -17 | 6843800 |
| -18 | 5763575 |
| -19 | 4592825 |
| -20 | 3413750 |
| -21 | 2124775 |
| -22 | 1128950 |
| -23 | 668850 |
| -24 | 214625 |
| -25 | 20175 |
| -26 | 0 |
