# Supplementary material for: The influence of thermal and hypoxia induced habitat compression on walleye (Sander vitreus) movements in a temperate lake
Source: Mov Ecol. 2025 Jan 7;13:1. doi: 10.1186/s40462-024-00505-6 (PMC11707865; doi:10.1186/s40462-024-00505-6)
Supplement: Supplementary file 4 [file 40462_2024_505_MOESM4_ESM.docx]

Table 3. Vemco VR2W acoustic receiver deployment locations, dates, depth, and the mean detection radius for stratified and isothermal periods (obtained from Wells et al. (50)).

| **Station** | **Receiver serial number** | **Latitude** | **Longitude** | **Deployment date** | **Final retrieval date** | **Depth**  **(m)** | **Mean detection radius (stratified)** | **Mean detection radius (isothermal)** |
| --- | --- | --- | --- | --- | --- | --- | --- | --- |
| 1 | 127832 | 43.30556 | -79.82056 | 7/8/2015 | 24/4/2019 | 16 | 267 | 425 |
| 2 | 127833 | 43.28639 | -79.79694 | 7/8/2015 | 16/4/2019 | 13 | 273 | 436 |
| 3 | 127836 | 43.29444 | -79.79972 | 7/8/2015 | 8/5/2019 | 12 | 276 | 440 |
| 4 | 127845 | 43.28806 | -79.83556 | 7/8/2015 | 15/11/2018 | 9 | 284 | 446 |
| 5 | 127849 | 43.28111 | -79.86472 | 7/8/2015 | 14/5/2019 | 4 | 322 | 408 |
| 6 | 127850 | 43.30666 | -79.80714 | 7/8/2015 | 8/5/2019 | 1 | 309 | 389 |
| 7 | 126879 | 43.30136 | -79.8401 | 8/8/2015 | 8/5/2019 | 8 | 285 | 387 |
| 8 | 126880 | 43.27515 | -79.79315 | 8/8/2015 | 14/5/2019 | 8 | 285 | 387 |
| 9 | 127827 | 43.28036 | -79.80383 | 8/8/2015 | 2/7/2020 | 12 | 276 | 440 |
| 12 | 127839 | 43.30048 | -79.80591 | 8/8/2015 | 29/6/2020 | 17 | 266 | 420 |
| 13 | 127846 | 43.29691 | -79.80157 | 7/8/2015 | 22/10/2019 | 8 | 296 | 366 |
| 15 | 127828 | 43.29361 | -79.85564 | 14/8/2015 | 14/5/2019 | 6 | 307 | 394 |
| 17 | 127837 | 43.29462 | -79.82189 | 18/8/2015 | 30/4/2019 | 7 | 286 | 383 |
| 17 | 547992 | 43.29482 | -79.82112 | 16/5/2019 | 2/6/2020 | 7 | 286 | 383 |
| 18 | 127840 | 43.2725 | -79.87839 | 18/8/2015 | 7/5/2019 | 8 | 278 | 375 |
| 19 | 127847 | 43.2778 | -79.85213 | 18/8/2015 | 7/5/2019 | 2 | 338 | 420 |
| 21 | 127841 | 43.28532 | -79.82618 | 20/8/2015 | 7/5/2019 | 1 | 297 | 435 |
| 22 | 127842 | 43.30178 | -79.79097 | 20/8/2015 | 16/5/2019 | 7 | 290 | 450 |
| 22 | 547988 | 43.30261 | -79.7904 | 17/5/2019 | 16/6/2020 | 7 | 290 | 450 |
| 23 | 127834 | 43.28511 | -79.868 | 29/8/2015 | 14/5/2019 | 5 | 315 | 400 |
| 25 | 127843 | 43.30979 | -79.81135 | 29/8/2015 | 8/5/2019 | 12 | 276 | 440 |
| 27 | 127829 | 43.27917 | -79.87444 | 11/11/2015 | 16/4/2019 | 6 | 332 | 422 |
| 28 | 126878 | 43.27582 | -79.88266 | 1/12/2015 | 6/5/2019 | 5 | 314 | 395 |
