# Supplementary material for: The influence of thermal and hypoxia induced habitat compression on walleye (Sander vitreus) movements in a temperate lake
Source: Mov Ecol. 2025 Jan 7;13:1. doi: 10.1186/s40462-024-00505-6 (PMC11707865; doi:10.1186/s40462-024-00505-6)
Supplement: Supplementary file 5 [file 40462_2024_505_MOESM5_ESM.docx]

Table 4. Number of monthly networks used in the full analysis.

| **Month** | **n** |
| --- | --- |
| April | 31 |
| May | 23 |
| June | 17 |
| July | 12 |
| August | 8 |
| September | 10 |
| October | 10 |
| November | 29 |
