# Supplementary material for: The influence of thermal and hypoxia induced habitat compression on walleye (Sander vitreus) movements in a temperate lake
Source: Mov Ecol. 2025 Jan 7;13:1. doi: 10.1186/s40462-024-00505-6 (PMC11707865; doi:10.1186/s40462-024-00505-6)
Supplement: Supplementary file 6 [file 40462_2024_505_MOESM6_ESM.docx]

Table 5. Number of networks per year.

| **Year** | **n** |
| --- | --- |
| 2016 | 61 |
| 2017 | 48 |
| 2018 | 31 |
