# Supplementary material for: The influence of thermal and hypoxia induced habitat compression on walleye (Sander vitreus) movements in a temperate lake
Source: Mov Ecol. 2025 Jan 7;13:1. doi: 10.1186/s40462-024-00505-6 (PMC11707865; doi:10.1186/s40462-024-00505-6)
Supplement: Supplementary file 7 [file 40462_2024_505_MOESM7_ESM.docx]

Table 6. Number of networks per walleye.

| **Walleye ID** | **n** | **Total number of detections** |
| --- | --- | --- |
| 15771 | 9 | 237024 |
| 15774 | 9 | 262436 |
| 83 | 8 | 296560 |
| 15763 | 7 | 218406 |
| 15772 | 7 | 208214 |
| 16062 | 7 | 95712 |
| 18969 | 7 | 325402 |
| 16051 | 6 | 318718 |
| 15755 | 5 | 256717 |
| 15765 | 5 | 220266 |
| 16059 | 5 | 174523 |
| 18967 | 5 | 348777 |
| 15759 | 4 | 277141 |
| 16052 | 4 | 325427 |
| 16055 | 4 | 277543 |
| 16056 | 4 | 212896 |
| 16057 | 4 | 298798 |
| 16058 | 4 | 294577 |
| 18965 | 4 | 110270 |
| 18973 | 4 | 153036 |
| 79 | 3 | 365282 |
| 14519 | 3 | 238243 |
| 15760 | 3 | 168143 |
| 15769 | 3 | 104175 |
| 16053 | 3 | 393931 |
| 16063 | 3 | 121650 |
| 18966 | 3 | 102648 |
| 14516 | 2 | 181638 |
| 16061 | 2 | 182838 |
| 15764 | 1 | 187876 |
| 15766 | 1 | 42947 |
| 18972 | 1 | 63157 |
