# Supplementary material for: The influence of thermal and hypoxia induced habitat compression on walleye (Sander vitreus) movements in a temperate lake
Source: Mov Ecol. 2025 Jan 7;13:1. doi: 10.1186/s40462-024-00505-6 (PMC11707865; doi:10.1186/s40462-024-00505-6)
Supplement: Supplementary file 8 [file 40462_2024_505_MOESM8_ESM.docx]

Table 7. Monthly distances for each individual walleye (not actual swimming distances, just a proxy for relative movement).

| **Walleye ID** | **Year** | **Month** | **Distance (km)** |
| --- | --- | --- | --- |
| 15755 | 2016 | April | 3481.6 |
| 15759 | 2016 | April | 2085.6 |
| 15760 | 2016 | April | 902.5 |
| 15763 | 2016 | April | 2893.6 |
| 15764 | 2016 | April | 701.7 |
| 15765 | 2016 | April | 845.3 |
| 15769 | 2016 | April | 3035.2 |
| 15771 | 2016 | April | 1198.6 |
| 15772 | 2016 | April | 1541.2 |
| 15774 | 2016 | April | 3567.3 |
| 79 | 2016 | April | 5339.2 |
| 83 | 2016 | April | 2264.7 |
| 15760 | 2016 | May | 75.3 |
| 15763 | 2016 | May | 724.7 |
| 15765 | 2016 | May | 264.1 |
| 15766 | 2016 | May | 727.0 |
| 15769 | 2016 | May | 258.7 |
| 15771 | 2016 | May | 166.9 |
| 15772 | 2016 | May | 1089.6 |
| 15774 | 2016 | May | 761.4 |
| 83 | 2016 | May | 564.3 |
| 15759 | 2016 | June | 594.6 |
| 15763 | 2016 | June | 1702.5 |
| 15769 | 2016 | June | 143.3 |
| 15774 | 2016 | June | 211.5 |
| 83 | 2016 | June | 1051.2 |
| 15763 | 2016 | July | 3617.8 |
| 15771 | 2016 | July | 52.2 |
| 15772 | 2016 | July | 189.9 |
| 15774 | 2016 | July | 157.0 |
| 18966 | 2016 | July | 102.8 |
| 18972 | 2016 | July | 185.4 |
| 18973 | 2016 | July | 661.1 |
| 83 | 2016 | July | 2329.4 |
| 15755 | 2016 | August | 989.9 |
| 15760 | 2016 | August | 3292.0 |
| 15765 | 2016 | August | 669.9 |
| 15771 | 2016 | August | 509.3 |
| 15772 | 2016 | August | 359.9 |
| 15755 | 2016 | September | 9180.0 |
| 15771 | 2016 | September | 192.2 |
| 15772 | 2016 | September | 1872.9 |
| 15774 | 2016 | September | 1299.9 |
| 18965 | 2016 | September | 3566.0 |
| 18973 | 2016 | September | 1687.9 |
| 83 | 2016 | September | 2660.9 |
| 15772 | 2016 | October | 2699.4 |
| 15774 | 2016 | October | 1405.7 |
| 83 | 2016 | October | 1470.8 |
| 15755 | 2016 | November | 2619.4 |
| 15759 | 2016 | November | 5354.1 |
| 15763 | 2016 | November | 2432.4 |
| 15765 | 2016 | November | 1760.9 |
| 15771 | 2016 | November | 1150.4 |
| 15772 | 2016 | November | 2883.8 |
| 15774 | 2016 | November | 3032.5 |
| 18965 | 2016 | November | 1703.4 |
| 18966 | 2016 | November | 2294.5 |
| 18967 | 2016 | November | 2600.0 |
| 79 | 2016 | November | 1987.9 |
| 83 | 2016 | November | 2024.4 |
| 15755 | 2017 | April | 889.1 |
| 15759 | 2017 | April | 913.6 |
| 15771 | 2017 | April | 1118.1 |
| 15774 | 2017 | April | 861.8 |
| 18965 | 2017 | April | 620.9 |
| 18967 | 2017 | April | 2444.4 |
| 18969 | 2017 | April | 1798.1 |
| 18973 | 2017 | April | 1015.7 |
| 79 | 2017 | April | 2791.8 |
| 83 | 2017 | April | 2993.0 |
| 15763 | 2017 | May | 2732.3 |
| 15765 | 2017 | May | 1189.4 |
| 16059 | 2017 | May | 4701.0 |
| 16062 | 2017 | May | 2519.5 |
| 16063 | 2017 | May | 6787.3 |
| 18966 | 2017 | May | 1363.2 |
| 18969 | 2017 | May | 2813.4 |
| 18973 | 2017 | May | 2489.7 |
| 15763 | 2017 | June | 5921.3 |
| 15771 | 2017 | June | 167.2 |
| 15774 | 2017 | June | 84.4 |
| 16056 | 2017 | June | 3057.9 |
| 16057 | 2017 | June | 707.7 |
| 16062 | 2017 | June | 302.9 |
| 16063 | 2017 | June | 3579.8 |
| 18969 | 2017 | June | 621.5 |
| 15771 | 2017 | July | 136.6 |
| 16056 | 2017 | July | 3029.4 |
| 16059 | 2017 | July | 325.6 |
| 16062 | 2017 | July | 589.5 |
| 16051 | 2017 | August | 423.7 |
| 16059 | 2017 | August | 708.1 |
| 16051 | 2017 | September | 8036.8 |
| 16051 | 2017 | October | 6987.5 |
| 16052 | 2017 | October | 4874.1 |
| 16053 | 2017 | October | 2308.1 |
| 16058 | 2017 | October | 7124.3 |
| 18967 | 2017 | October | 1572.4 |
| 16051 | 2017 | November | 7343.3 |
| 16052 | 2017 | November | 7373.0 |
| 16053 | 2017 | November | 5720.0 |
| 16056 | 2017 | November | 1887.0 |
| 16058 | 2017 | November | 6441.0 |
| 16059 | 2017 | November | 7765.0 |
| 16061 | 2017 | November | 3640.6 |
| 16062 | 2017 | November | 4806.9 |
| 18965 | 2017 | November | 163.6 |
| 18969 | 2017 | November | 5409.9 |
| 16051 | 2018 | April | 1087.3 |
| 16052 | 2018 | April | 826.4 |
| 16055 | 2018 | April | 181.5 |
| 16057 | 2018 | April | 850.5 |
| 16058 | 2018 | April | 2078.4 |
| 16062 | 2018 | April | 979.0 |
| 16063 | 2018 | April | 2797.7 |
| 18967 | 2018 | April | 1438.2 |
| 18969 | 2018 | April | 2084.4 |
| 16053 | 2018 | May | 1579.1 |
| 16055 | 2018 | May | 37.1 |
| 16057 | 2018 | May | 246.3 |
| 16061 | 2018 | May | 222.5 |
| 16062 | 2018 | May | 267.6 |
| 18969 | 2018 | May | 412.7 |
| 16055 | 2018 | June | 1155.6 |
| 16058 | 2018 | June | 83.0 |
| 16059 | 2018 | June | 152.9 |
| 16062 | 2018 | June | 438.8 |
| 14519 | 2018 | August | 1003.4 |
| 14516 | 2018 | September | 849.3 |
| 14519 | 2018 | September | 542.5 |
| 14516 | 2018 | October | 1557.2 |
| 18967 | 2018 | October | 2317.5 |
| 14519 | 2018 | November | 2046.0 |
| 16051 | 2018 | November | 2970.4 |
| 16052 | 2018 | November | 7712.8 |
| 16055 | 2018 | November | 2547.8 |
| 16056 | 2018 | November | 944.5 |
| 16057 | 2018 | November | 2089.7 |
| 18969 | 2018 | November | 4162.0 |
