# Supplementary material for: The influence of thermal and hypoxia induced habitat compression on walleye (Sander vitreus) movements in a temperate lake
Source: Mov Ecol. 2025 Jan 7;13:1. doi: 10.1186/s40462-024-00505-6 (PMC11707865; doi:10.1186/s40462-024-00505-6)
Supplement: Supplementary file 9 [file 40462_2024_505_MOESM9_ESM.docx]

**Table 8. Habitat categories per year, including daily minimum, maximum, and mean proportions (%) and the cumulated proportion of harbour for the full season between 27 May and 02 October each year.**

| Year | Habitat | Daily min (%) | Daily mean (%) | Daily max (%) | Cumulative proportion Season |
| --- | --- | --- | --- | --- | --- |
| 1976 | Hypoxic | 1.15 | 29.26 | 59.59 | 0.22 |
| 1976 | Optimum | 7.36 | 41.18 | 63.50 | 0.34 |
| 1976 | Suitable | 6.28 | 44.72 | 100.00 | 0.45 |
| 1977 | Hypoxic | 1.46 | 26.05 | 59.59 | 0.25 |
| 1977 | Optimum | 6.54 | 42.86 | 58.11 | 0.35 |
| 1977 | Suitable | 5.39 | 41.91 | 100.00 | 0.42 |
| 1978 | Hypoxic | 1.46 | 26.47 | 60.78 | 0.23 |
| 1978 | Optimum | 6.54 | 37.83 | 58.11 | 0.36 |
| 1978 | Suitable | 5.39 | 42.21 | 100.00 | 0.42 |
| 1979 | Hypoxic | 2.67 | 35.67 | 65.86 | 0.35 |
| 1979 | Optimum | 6.54 | 38.28 | 63.50 | 0.35 |
| 1979 | Suitable | 5.68 | 30.41 | 100.00 | 0.31 |
| 1987 | Hypoxic | 0.31 | 32.27 | 59.59 | 0.29 |
| 1987 | Optimum | 6.28 | 39.15 | 58.11 | 0.37 |
| 1987 | Suitable | 5.39 | 33.91 | 100.00 | 0.34 |
| 1988 | Hypoxic | 0.08 | 30.90 | 65.86 | 0.30 |
| 1988 | Optimum | 6.41 | 33.05 | 52.43 | 0.22 |
| 1988 | Suitable | 6.11 | 47.69 | 100.00 | 0.48 |
| 1989 | Hypoxic | 1.46 | 30.56 | 53.48 | 0.28 |
| 1989 | Optimum | 6.11 | 35.45 | 58.11 | 0.31 |
| 1989 | Suitable | 5.68 | 41.99 | 100.00 | 0.42 |
| 1990 | Hypoxic | 0.71 | 21.51 | 53.48 | 0.17 |
| 1990 | Optimum | 7.36 | 47.87 | 68.44 | 0.39 |
| 1990 | Suitable | 4.63 | 44.60 | 100.00 | 0.45 |
| 1994 | Hypoxic | 0.08 | 21.47 | 36.50 | 0.15 |
| 1994 | Optimum | 14.47 | 41.02 | 52.43 | 0.33 |
| 1994 | Suitable | 11.07 | 52.47 | 100.00 | 0.53 |
| 1997 | Hypoxic | 0.71 | 25.96 | 53.48 | 0.17 |
| 1997 | Optimum | 12.69 | 46.13 | 73.07 | 0.39 |
| 1997 | Suitable | 4.34 | 45.04 | 100.00 | 0.45 |
| 1998 | Hypoxic | 1.46 | 23.37 | 53.48 | 0.24 |
| 1998 | Optimum | 6.28 | 49.83 | 68.44 | 0.42 |
| 1998 | Suitable | 4.63 | 34.85 | 98.54 | 0.35 |
| 1999 | Hypoxic | 1.46 | 32.04 | 59.59 | 0.28 |
| 1999 | Optimum | 6.41 | 44.17 | 68.44 | 0.41 |
| 1999 | Suitable | 4.63 | 31.77 | 100.00 | 0.32 |
| 2000 | Hypoxic | 0.08 | 23.52 | 41.89 | 0.14 |
| 2000 | Optimum | 12.38 | 52.55 | 68.44 | 0.40 |
| 2000 | Suitable | 4.94 | 46.50 | 100.00 | 0.47 |
| 2001 | Hypoxic | 1.46 | 28.58 | 47.57 | 0.28 |
| 2001 | Optimum | 5.91 | 42.93 | 63.50 | 0.36 |
| 2001 | Suitable | 4.94 | 36.81 | 100.00 | 0.37 |
| 2002 | Hypoxic | 0.08 | 27.49 | 49.43 | 0.22 |
| 2002 | Optimum | 6.11 | 39.90 | 68.44 | 0.33 |
| 2002 | Suitable | 4.94 | 45.50 | 100.00 | 0.46 |
| 2003 | Hypoxic | 0.31 | 16.11 | 50.81 | 0.07 |
| 2003 | Optimum | 12.69 | 39.90 | 58.11 | 0.31 |
| 2003 | Suitable | 16.31 | 62.49 | 100.00 | 0.63 |
| 2004 | Hypoxic | 0.08 | 14.79 | 31.56 | 0.12 |
| 2004 | Optimum | 14.47 | 56.74 | 77.40 | 0.51 |
| 2004 | Suitable | 4.05 | 37.97 | 100.00 | 0.38 |
| 2005 | Hypoxic | 0.08 | 12.05 | 26.93 | 0.10 |
| 2005 | Optimum | 7.36 | 37.98 | 68.44 | 0.37 |
| 2005 | Suitable | 8.96 | 53.75 | 100.00 | 0.54 |
| 2006 | Hypoxic | 0.08 | 9.93 | 36.50 | 0.08 |
| 2006 | Optimum | 7.36 | 46.38 | 77.40 | 0.42 |
| 2006 | Suitable | 11.01 | 49.73 | 100.00 | 0.50 |
| 2007 | Hypoxic | 0.08 | 12.31 | 31.56 | 0.10 |
| 2007 | Optimum | 12.38 | 48.98 | 77.40 | 0.45 |
| 2007 | Suitable | 8.96 | 45.60 | 100.00 | 0.46 |
| 2008 | Hypoxic | 0.08 | 16.32 | 41.89 | 0.11 |
| 2008 | Optimum | 21.18 | 48.50 | 77.40 | 0.44 |
| 2008 | Suitable | 4.94 | 45.92 | 100.00 | 0.46 |
| 2009 | Hypoxic | 0.31 | 16.97 | 36.50 | 0.13 |
| 2009 | Optimum | 27.73 | 51.51 | 73.07 | 0.43 |
| 2009 | Suitable | 8.39 | 44.68 | 100.00 | 0.45 |
| 2010 | Hypoxic | 0.31 | 20.32 | 41.89 | 0.17 |
| 2010 | Optimum | 6.28 | 39.91 | 68.44 | 0.33 |
| 2010 | Suitable | 4.63 | 49.95 | 99.69 | 0.50 |
| 2011 | Hypoxic | 0.08 | 16.62 | 41.89 | 0.14 |
| 2011 | Optimum | 7.36 | 42.93 | 73.07 | 0.39 |
| 2011 | Suitable | 5.68 | 47.63 | 100.00 | 0.48 |
| 2012 | Hypoxic | 0.31 | 13.78 | 41.89 | 0.09 |
| 2012 | Optimum | 11.59 | 42.20 | 73.07 | 0.39 |
| 2012 | Suitable | 12.08 | 52.89 | 100.00 | 0.53 |
| 2013 | Hypoxic | 0.08 | 15.29 | 36.50 | 0.13 |
| 2013 | Optimum | 14.47 | 45.60 | 63.50 | 0.37 |
| 2013 | Suitable | 9.57 | 50.70 | 100.00 | 0.51 |
| 2014 | Hypoxic | 0.08 | 13.88 | 41.89 | 0.12 |
| 2014 | Optimum | 14.47 | 46.06 | 58.11 | 0.40 |
| 2014 | Suitable | 10.33 | 48.25 | 100.00 | 0.49 |
| 2015 | Hypoxic | 0.08 | 18.37 | 53.48 | 0.16 |
| 2015 | Optimum | 6.41 | 43.87 | 63.50 | 0.37 |
| 2015 | Suitable | 4.34 | 47.48 | 100.00 | 0.47 |
| 2016 | Hypoxic | 0.08 | 15.24 | 36.50 | 0.11 |
| 2016 | Optimum | 12.02 | 39.19 | 77.40 | 0.39 |
| 2016 | Suitable | 5.39 | 50.15 | 100.00 | 0.51 |
| 2017 | Hypoxic | 8.39 | 26.45 | 47.25 | 0.17 |
| 2017 | Optimum | 27.73 | 51.48 | 63.50 | 0.46 |
| 2017 | Suitable | 6.23 | 37.16 | 100.00 | 0.37 |
| 2018 | Hypoxic | 0.08 | 12.69 | 36.50 | 0.10 |
| 2018 | Optimum | 5.91 | 41.09 | 91.26 | 0.40 |
| 2018 | Suitable | 8.03 | 50.10 | 100.00 | 0.50 |
| 2019 | Hypoxic | 0.31 | 5.01 | 8.74 | 0.02 |
| 2019 | Optimum | 14.47 | 45.55 | 73.07 | 0.38 |
| 2019 | Suitable | 25.24 | 59.92 | 100.00 | 0.60 |
