# Supplementary figures and images for: The influence of thermal and hypoxia induced habitat compression on walleye (Sander vitreus) movements in a temperate lake
Source: Mov Ecol. 2025 Jan 7;13:1. doi: 10.1186/s40462-024-00505-6 (PMC11707865; doi:10.1186/s40462-024-00505-6)

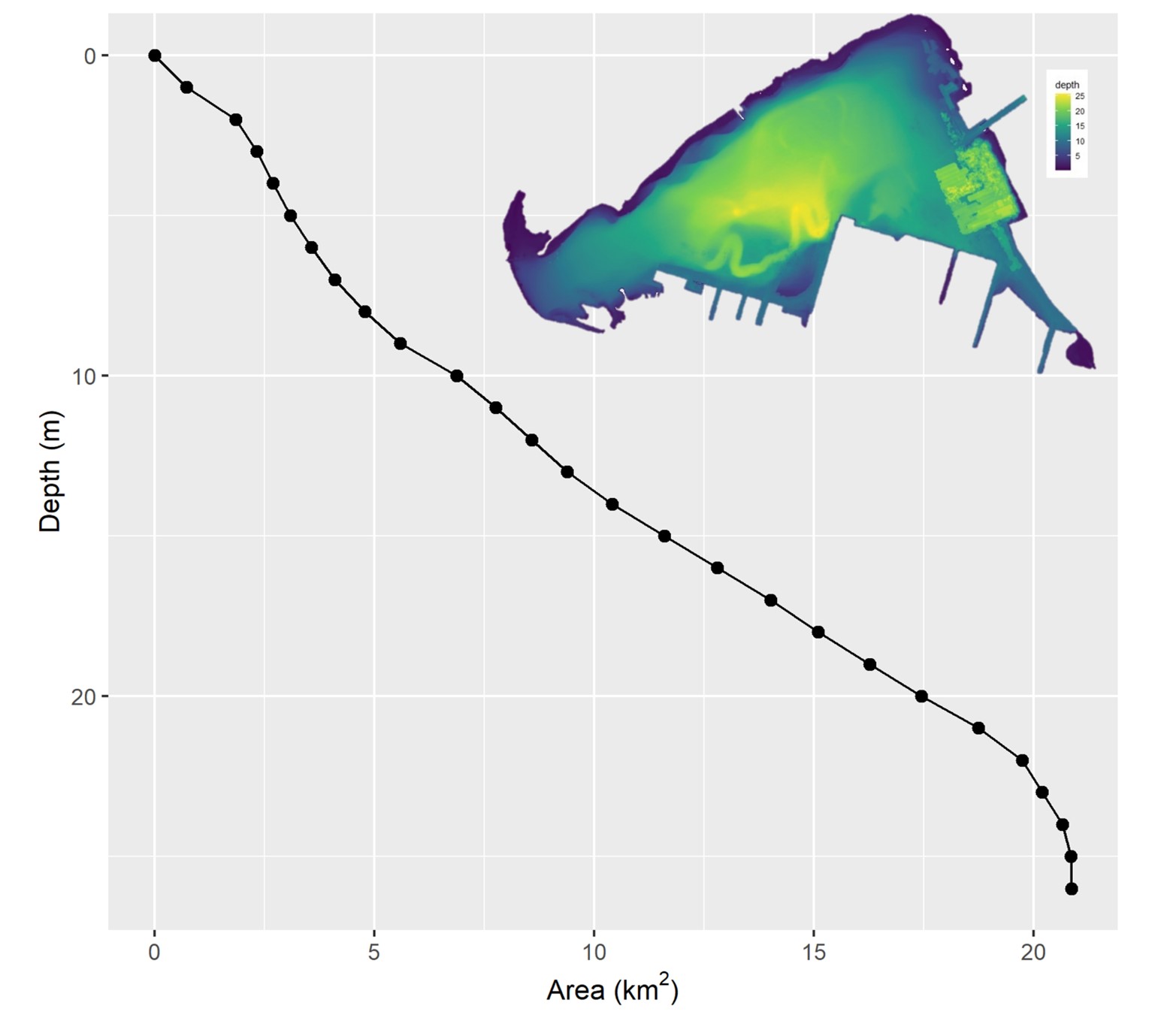

Supplement: Supplementary file 10 [file 40462_2024_505_MOESM10_ESM.jpg]

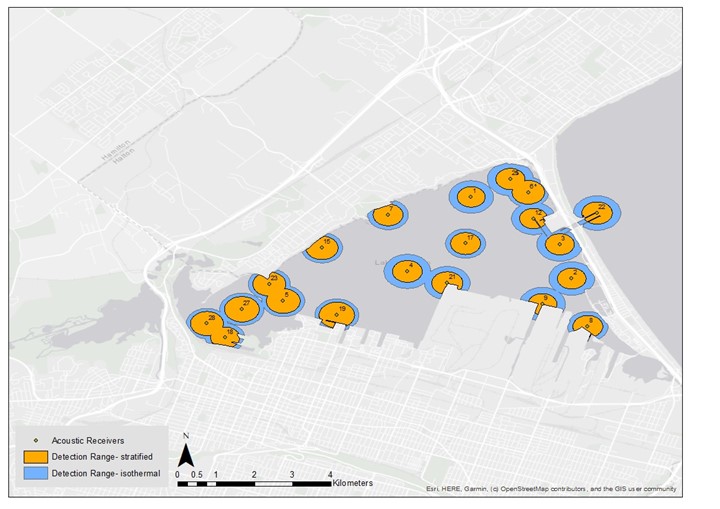

Supplement: Supplementary file 11 [file 40462_2024_505_MOESM11_ESM.jpg]

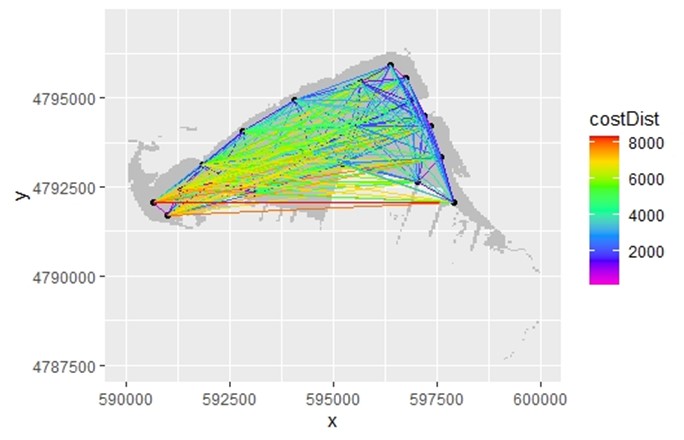

Supplement: Supplementary file 12 [file 40462_2024_505_MOESM12_ESM.jpg]

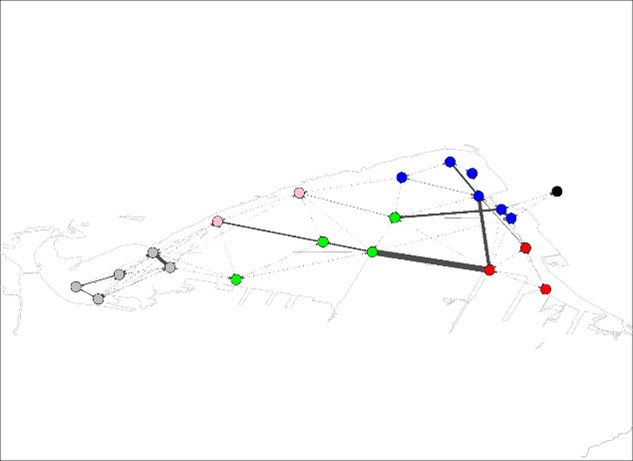

Supplement: Supplementary file 13 [file 40462_2024_505_MOESM13_ESM.jpg]

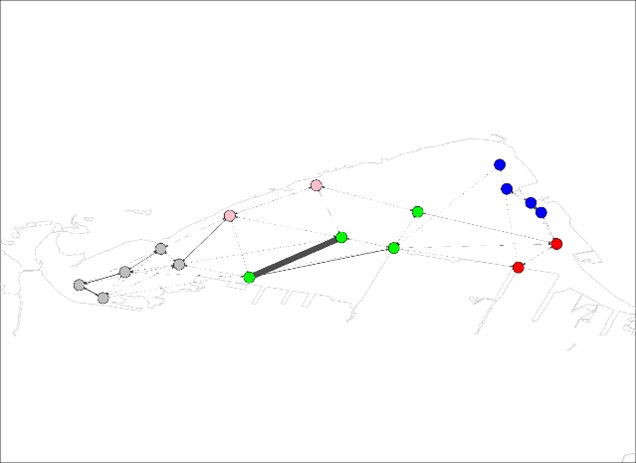

Supplement: Supplementary file 14 [file 40462_2024_505_MOESM14_ESM.jpg]
