# Supplementary material for: The influence of thermal and hypoxia induced habitat compression on walleye (Sander vitreus) movements in a temperate lake
Source: Mov Ecol. 2025 Jan 7;13:1. doi: 10.1186/s40462-024-00505-6 (PMC11707865; doi:10.1186/s40462-024-00505-6)
Supplement: Supplementary file 20 [file 40462_2024_505_MOESM20_ESM.docx]

Supplementary Materials

Figures

Figure 1. Hypsographic curve obtained from the Digital Elevation Model of Hamilton Harbour (excluding Cootes Paradise). Digital Elevation Model of Hamilton Harbour, excluding Cootes Paradise as walleye have not been documented to use the marsh area.

Figure 2. Map of the array with the mean detection range during the stratified (orange) and isothermal (blue) periods (data obtained from Wells et al. 2021).

Figure 3. Least cost path calculations (m) between each pair of receivers in the 21-station array. Although lines show paths over land, the distances were calculated ‘as the fish swims’, i.e., around the land border.

Figure 4. Network for walleye #83 in a) September 2016 and b) June 2016. Thickness of connecting lines indicates the number of movements between those two nodes (receiver stations). Node colours indicate geographic region of the Harbour.

Figure 5. Categorized abiotic conditions per 1 m depth for each day of the year for every year data were available (one panel per decade). Black shading is hypoxic (<3 mg/L), green represents physiologically optimal for walleye (Sander vitreus; 18 – 23 °C, > 5 mg/L), and white is suitable (all temperatures, > 3 mg/L).
